# Innovations in pavement design and engineering: A 2023 sustainability review

**DOI:** 10.1016/j.heliyon.2024.e33602

**Published:** 2024-06-25

**Authors:** Jaime Styer, Lori Tunstall, Amy Landis, James Grenfell

**Affiliations:** a Department of Engineering Design and Society, Humanitarian Engineering and Science Program, Colorado School of Mines, 1500 Illinois St, Golden, CO, 80401, USA; b Department of Civil and Environmental Engineering, Colorado School of Mines, 1500 Illinois St, Golden, CO, 80401, USA; c Sustainable Infrastructure Materials, Australian Road Research Board, 80a Turner Street, Port Melbourne, VIC, 3207, Australia

**Keywords:** Sustainable pavement, Triple bottom line sustainability, Pavement innovation, Socio-technical design

## Abstract

Transportation infrastructure is essential to a nation's everyday life and economic activity. Accordingly, pavement design and engineering are imperative to ensure safe, comfortable, and efficient transportation of goods, services, and people across countries. Pavements should be designed to be adaptable to changing traffic inputs and environmental conditions and always strive to fulfill the requirements of the end-users, including safety, durability, comfort, efficiency, sustainability, and cost. This review highlights innovations in paving technologies with a focus on sustainability from a socio-technical perspective; the scope is meant to be comprehensive but not exhaustive. The discussion categorizes paving design and technology innovations into two high-level sections: 1) high-volume urban pavement innovations and 2) low-volume rural pavement innovations.

## 1 Introduction

Roads are a crucial part of transportation infrastructure [1]. Roads connect communities and provide access to employment, education, medicine, and other vital services. They also support economic development by enabling trade and commerce. The efficacy of roads to perform these essential roles largely depends on their pavement design, which affects the type of loads that can be transported, how long the paved roads will last, the environmental and economic impact to communities, and more. While the construction industry is an essential source of income for many countries and important for social and economic development, it often contributes to many secondary environmental and social issues, particularly in rapidly developing communities.

Innovations in paving design and engineering have arisen for many reasons, such as new challenges presented by changing traffic and environmental conditions, the desire for decreased cost and increased longevity, and increasing collaboration across the globe. Societal and environmental pressures for industries to become more sustainable and responsible have also sparked innovation in the pavement industry. The effects of unsustainable processes and activities from industries can be seen at multiple echelons across the globe. Most notable are the negative environmental impacts, such as climate change, pollution, exhaustion of nonrenewable resources, increasing waste generation, biodiversity loss, and more. According to the United Nations Environment Program, the whole buildings and construction sector accounted for 38 % of global energy-related CO_2_ emissions in 2019 [2]. Along with the environmental push, transportation agencies and the public are also driving industries to be more sustainable in their practices [3].

The global struggle to address climate change has prompted many questions, such as who is responsible and who should fix it. At present, when examining annual CO_2_ emissions, Asia is by far the largest emitter, accounting for around half of global emissions. North America is next at 18 % of global emissions, followed closely by Europe at 17 %, while Africa and South America independently account for 3–4% [4]. Historically, however, the global north, most notably the United States and European Union, has been responsible for the majority of contributions to cumulative CO_2_ emissions [5]. While opinions may differ on who is primarily responsible for climate change, as the former Senior Fellow at the Center for Global Development states, decarbonization is everyone's responsibility [6]. Further, when discussing the effects of climate change and related environmental issues, it would be harmful not to acknowledge that inequality shapes the impacts of climate change [7]. For example, populations that have contributed least to climate change through their emissions, such as low-income countries, are likely the most vulnerable to its effects [7]. To address this inequality, special consideration should be given to those more vulnerable to the effects of climate change when responding to societal pressures to become more sustainable and responsible. These problems demonstrate the importance of industries innovating and becoming more sustainable and responsible. However, for innovative, sustainable solutions to be effective, it is vital for designers to not only conceive sustainable innovations but also understand the contextual conditions of the implementation site and identify the most appropriate implementation and scaling-up strategies [8].

Understanding the socio-technical context is essential to ensure the sustainability of engineering and design projects, particularly when considering solutions designed for populations outside the innovator's cultural context, since devaluing local knowledges, skills, and beliefs often leads to the failure of engineering projects. For example, one expert concludes that the ultimate failure of the Tanzania Ujamaa Village Campaign was due to the project planners' outsider designs that did not consider larger contexts or local knowledges [9]. The Tanzania Ujamaa Village Campaign was a large-scale social engineering attempt made by officials in the central government to permanently settle most of the country's population in “modern” villages. Everything about the villages was planned, partly or wholly, by government officials who (1) had complete faith in what they took for “modern agriculture” and (2) had an underlying conviction that “the peasants did not know what was good for them” [9]. Ultimately, this project took skilled people and put them in a setting where their skills were of little use [9]. For example, almost 60 % of the new “modern” villages were on semiarid land not suitable for long-term cultivation; additionally, the regulated labor plans bore no relation to the seasonal supply of local labor or local peoples' own goals [9]. According to the author, “the failure of ujamaa villages was almost guaranteed by the high modernist hubris of planners and specialists who believed that they alone knew how to organize a more satisfactory, rational, and productive life for their citizens” [9]. To develop a solution of best fit, technical and social variables must be considered, such as local availability of materials and technologies, local cultural norms, local laws and regulations, local economic capabilities, and more [10]. In this sense, Jamshidi et al. state a pavement system must “be constructed based on local materials, construction technologies, available financial sources, and social norms” [10].

Beginning with a discussion on the importance of framing pavement projects from a community-based, socio-technical perspective, this work reviews, summarizes, and categorizes recent paving design and engineering innovations within two high-level sections: 1) high-volume urban pavement innovations and 2) low-volume rural pavement innovations. The high-volume urban innovations section is separated into three categories. First, significant innovations in the primary bound pavement types, rigid and flexible pavements, are described. Then innovations in smart and multifunctional pavements are highlighted. After this, the low-volume rural pavement innovations section is divided into two subsections 1) unbound granular pavements and 2) stabilized pavements.

### 1.1 Brief pavement overview

**Fig. 1:**
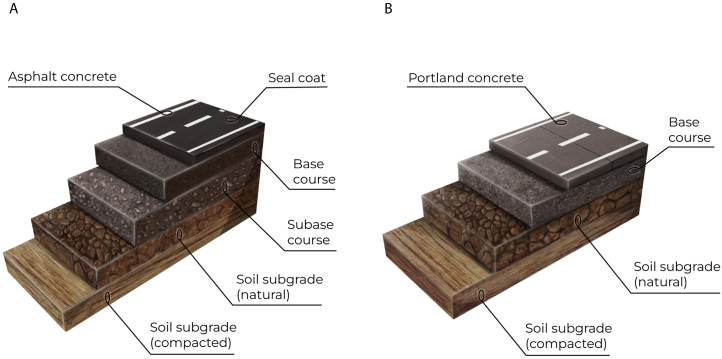
Flexible (A) and Rigid (B) pavement cross-sections.

Bound pavements can be categorized into three primary types – flexible (asphalt), rigid (concrete), and composite [11]. Flexible pavements typically consist of a subgrade (compacted soil) on the bottom, topped with granular subbase/base layers, and asphalt concrete with a seal coat or wearing course on top (Fig. 1A). Flexible pavements can also have sprayed seals and interlocking concrete block pavers as surface layers [12]. The “flexible” namesake derives from how the asphalt ideally transmits uniform stresses and nonuniform deflections to the underlying layers. Rigid pavements typically consist of a Portland concrete layer, with transverse joints at prescribed intervals, placed over a subgrade and a granular base layer (Fig. 1B). Sometimes, however, the Portland concrete layer is placed directly over the subgrade, and the base layer is excluded. Contrary to flexible pavements, rigid pavements are designed to transmit nonuniform stresses and uniform deflections. In other words, the deflection of a rigid pavement should be relatively consistent and very small due to the thick concrete slab top layer and its high stiffness, which effectively distributes loads throughout the slab area [11,13]. Composite pavements utilize both asphalt and concrete and are typically the product of pavement rehabilitation.

Unbound granular pavements can be sealed or unsealed. , granular pavements is often achieved using bituminous seals and slurries and requires the placement and compaction of the unbound pavement materials to ensure a uniform surface free from loose, segregated, and contaminated areas [14]. However, as most unbound pavements are also unsealed [15], we focus our discussion on unsealed unbound pavements. According to the Australian Road Research Board (ARRB) Group, there are three types of unsealed roads including (1) unformed roads, or non-engineered roads; (2) formed roads, designed earth roads made of local materials, and (3) formed and graveled roads, which are made from imported granular material [15]. Unsealed roads contribute to many significant domains, including providing access to rural communities and facilitating access for these communities to essential services such as healthcare, education and local markets [16,17]; moving primary produce to markets; moving within state forests and defense training areas, including fire management; providing access to forests or fire management on public lands; providing access to haulage roads for the mining and timber industries; as well as recreational, social, and tourist pursuits [15].

### 1.2 Unpacking sustainability

**Fig. 2:**
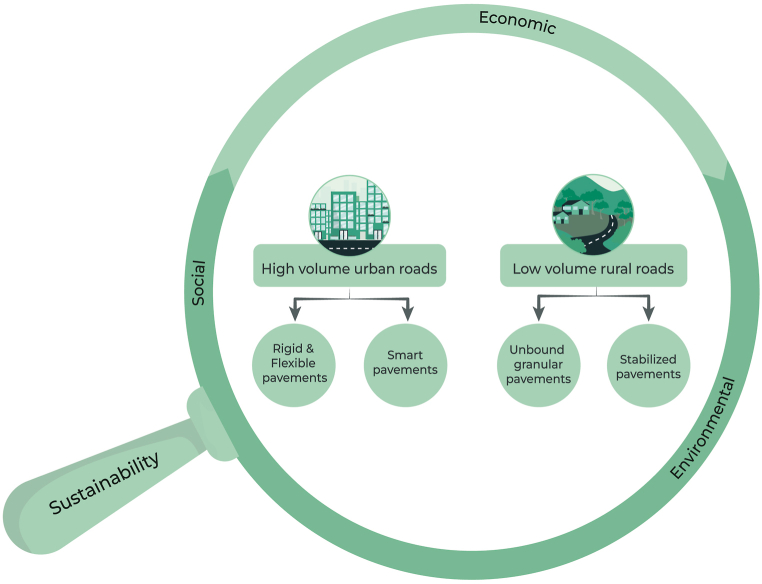
Analyzing innovations in pavement technologies through the “lens” of triple bottom line sustainability.

Although many of the discussions regarding sustainability are focused on environmental sustainability, sustainability is an extensive term comprising much more. For example, Crane & Matten define sustainability as "the long-term maintenance of systems according to environmental, economic, and social considerations" ([18], p. 32). Within this definition of sustainability, the critical framework many corporations utilize is known as the Triple Bottom Line, created by John Elkington in 1994 [19]. This triple bottom line sustainability framework analyzes a business’s economic, social, and environmental impact; however, as Elkington points out, over time this framework has been simplified into an accounting tool, deviating from its intended purpose [19]. The definition of sustainability is intentionally broad, as its inherent goal is to revolutionize how companies think about their business practices. Thus, sustainability cannot and should not be simplified into a checkbox for industries but rather must be adopted as an essential mindset behind every decision and innovation in any industry—in other words, the lens through which everything is evaluated (Fig. 2) [19].

In the transportation sector and pavement industry, sustainable design objectives should aim at “environmental awareness and compliance, simultaneously adapting to economic, budgetary limitations while at the same time also fulfilling the emerging societal needs and demands” ([20], p. 541). Although this definition of sustainability is broad, as Van Dam et al. argue, sustainability is context-sensitive, and “it is important to recognize that, in some cases, it may even be counterproductive to try to introduce certain features that are thought to be sustainable without a complete assessment” [21]. For example, in the context of pavement design, utilizing local aggregate that is readily available and meets local requirements could be a better environmental decision when compared to recycled materials that need to be transported a great distance [21]. Since each situation is unique, understanding the local context of where pavement is to be placed, including factors such as the local availability of materials, local maintenance capacity, climate considerations, and more, is essential to its sustainability.

### 1.3 Sustainability tools

Many tools exist to aid in quantifying the three pillars of sustainability—environmental, economic, and social [19]. A few that have been applied to pavements include life cycle assessment (LCA), techno-economic analysis (TEA), and material flow analysis (MFA). Carbon footprinting is a subset of LCA. Social-LCA is a tool that has not been applied to pavement systems but is useful for understanding the social pillar. LCAs quantify the environmental impacts of a product, process, or system over its entire life cycle, from raw materials acquisition to end of life. LCA methods are defined by the ISO 14040 series (ISO 2006). LCAs often follow the Product Category Rules (PCRs) that have been published for the particular product type: PCRs exist for cements [[22], [23], [24]] and concrete [25]. Carbon footprints follow LCA methods, but whereas LCA tracks all environmental flows, a carbon footprint only tracks greenhouse gases. TEA is often used early in the design stages of new product development to elucidate economic and design hurdles [26]. More recent and sophisticated approaches to TEA expand the tool to incorporate market size, policy incentives, and criticality of supply chains [27]. TEA helps to assess commercial availability of equipment and feedstocks. There is no TEA methodology standard, but methods typically follow the first several steps of an LCA, and they can be conducted in parallel. Often TEAs follow methods described by the Department of Energy [26]. Materials flow analysis (MFA), also known as substance flow analysis (SFA) when referring to a specific substance like asphalt, is a method based on the law of mass conservation for quantifying stocks and flows of goods or substances through the economy [28]. Results are typically displayed as a Sankey diagram and show the mass flows of materials through an economy. MFA elucidates where the largest flows, losses, and accumulation of materials occur within systems. MFA is particularly helpful to evaluate opportunities for circular economy solutions. Finally, Social-LCA (*S*-LCA) is the broad term for a set of tools that assess social impacts of a product, process, or system following similar methods to LCA. These tools have social impact indicator databases that contain inventories of geography-specific supply chain data that identify social impacts or risks for a wide range of stakeholders and manufacturing processes [29].

There have been a handful of specific sustainability tools developed for pavements and roads, including the U.S. Federal Highway Administration's (FHWA) pavement LCA tool [30], U.S. Department of Transportation's (DOT) Infrastructure Voluntary Evaluation Sustainability Tool (INVEST) [31], the Sustainability Assessment Tool For Pavements (SAT4P) developed by ARRB and the National Asset Center of Excellence (NACOE) [32], as well as Greenroads [33]. Other tools more broadly focus on infrastructure sustainability design, construction, and management tools that could be used in pavement design, such as the Envision rating system [34] and Australia's infrastructure sustainability tool [35]. There is one LCA-specific tool developed by FHWA, LCA Pave, which is a spreadsheet-based LCA tool to assess environmental impacts of pavement material and design decisions [36].

Most of the available tools are rating systems that aim to deliver more sustainable roadways using a rating system often used for certification, such as the Infrastructure Sustainability Council of Australia's IS Rating scheme, the Greenroads Foundation's Greenroads Rating System, BE2ST-in-Highways, and GreenLITES; a review of these rating systems was conducted by Mattinzioli et al. [37]. Some of these rating systems are third-party, while others are self-assessments. Rating systems award points for sustainable design and construction practices and can be used to certify projects. They are used by roadway projects to evaluate and deliver sustainable transportation infrastructure, and studies show that rating systems such as Greenroads result in roads with reduced costs (both initial and long-term) and environmental impacts [38].

DOT published the Infrastructure Voluntary Evaluation Sustainability Tool (INVEST) as a part of the Sustainable Highways Initiative. INVEST is a web-based self-evaluation toolkit that guides transportation agencies through sustainability best practices for their projects and programs. The toolkit covers the full life cycle (but is not an LCA Tool) of transportation services, including system planning, project planning, design, and construction, and operations and maintenance. DOT developed INVEST for voluntary use by transportation agencies to assess and enhance the sustainability of their projects and programs.

This paper reviews innovations in pavement design and engineering. While many of the innovations discussed in this paper offer several advantages and claim contributions to sustainability, it is important to note that they may not fit the needs of every context. Thus, before implementing new pavement designs, it is essential to work with local communities to understand the socio-technical context of desired implementation locations.

## 2 Materials and methods

This review highlights innovations in paving technologies with a focus on sustainability from a socio-technical perspective; the scope is meant to be comprehensive but not exhaustive. For this study a narrative scoping literature review method was employed to ensure a broad overview of paving technologies and recent innovations. Utilizing a more flexible research protocol allowed the review to explore a more diverse and extensive set of literature. Although multiple search terms were utilized throughout the review process, citation chaining and resource sharing methods were also employed to investigate additional relevant academic sources, thus the search terms do not entirely summarize the scope of the review. Despite this, some of the search terms utilized include “paving design”, “paving materials”, “pavement design and materials review”, “pavement design and materials innovation”, “paving technology review”, “sustainable pavement”, “sustainable pavement review”, “state of the art pavement”, “pavement” and “sustainability”, as well as others detailing the specific paving technologies and designs discussed in this review.

Although the main literature item type investigated in this review is journal articles, relevant conference papers, reference documents, academic magazine articles, governmental webpages, books, reports, and theses/dissertations were also collected and analyzed. The main database utilized to collect these items was Google Scholar, however, EBSCO and ProQuest were also used. In addition, multiple items reviewed were shared by research collaborators, academic advisors, and subject matter experts to ensure a more comprehensive review. Overall, 221 studies were reviewed for the present study. The discussion then categorizes these into two high-level sections: 1) high-volume urban pavement innovations and 2) low-volume rural pavement innovations.

## 3 Results

To facilitate the evaluation of traditional and emerging pavement technologies, a summary of the main innovations discussed in this review is provided in Table 1. For each innovation, a brief synopsis of the technology is provided, in addition to the potential benefits of the innovation and its barriers for adoption.

**Table 1:** Pavement innovation summary.

Innovation Name	Section	Synopsis	Benefits	Barriers	References
Supplementary Cementitious Materials	Rigid	Materials used to reduce CO_2_ emissions by partially replacing ordinary Portland cement (OPC); traditionally industrial waste products	Reduction of CO_2_ emissions; increased resistance to deterioration; improved long-term compressive strength; reduced costs	Nontraditional materials require further research to determine impact on pavement properties and reactivity times	[39,[40], [41], [42], [43], [44], [45]]
Alternative Low-Carbon Binders	Rigid	Used to reduce CO_2_ emissions by replacing OPC with alternative low-carbon cements, such as calcium sulfoaluminate clinker	Reduction in CO_2_ emissions	Uncertainty regarding long-term durability; perceptions of high costs; fear of unknown	[39,46,47]
Recycled Material Aggregates	Rigid	Recycled materials used to replace aggregates. Some common examples include reclaimed asphalt pavement (RAP) and recycled concrete aggregate (RCA)	Reduction in landfill waste, contribution to the circular economy, reducing dependency on nonrenewable resources	May require treatment to ensure performance; may pose a technical risk or maintenance liability; dependent upon locally available materials	[1,11,12,20,21,[48], [49], [50], [51], [52], [53], [54], [55], [56], [57], [58]]
Precast Concrete Pavement Systems	Rigid	Precast concrete panels are manufactured and cured at an external location then brought to the construction site, where they are installed	Minimal weather restrictions when placing; fast construction times; better quality concrete	Much higher initial cost; load transfer issues created between the panels and existing pavement; require careful leveling during placement	[59,60]
Ultra-High Performance Concrete Overlays	Rigid	Concrete with a dense granular matrix that is fiber-reinforced and exhibits ultra-enhanced durability and mechanical properties	Ultra-high compressive strength; extremely high impermeability; negligible drying shrinkage if properly cured; excellent post-cracking tensile capacity; high early strength; fast construction times	Perception of reduced environmental sustainability; increased cost due to high usage of OPC and silica fume	[[61], [62], [63], [64], [65], [66], [67], [68], [69], [70]]
Self-Compacting Concrete	Rigid	A high-strength and high-performance concrete that does not necessitate vibration due to its lowered water-cement ratio and higher mortar percentage	Superior durability characteristics; improved workability; high strength; faster construction and reduced traffic closure time; reduced need for vibration equipment and reduced noise emission	Susceptible to numerous forms of cracking and other structural defects; requires solids to stay well dispersed in fluid; not environmentally friendly currently	[[71], [72], [73], [74], [75], [76], [77], [78], [79], [80], [81], [82], [83], [84]]
Warm-Mix Asphalt (WMA)	Flexible	Asphalt that is produced and placed at temperatures between 100 and 140 °C.	Low energy consumption; decreased environmental degradation and allows higher proportions of recycled materials; improved health and safety conditions; extended paving window; improved physical and mechanical properties, durability, workability and compaction efficiency	Increased susceptibility to trapped moisture causing premature pavement decay	[3,20,85,[86], [87], [88], [89], [90]]
Cold-Mix Asphalt (CMA)	Flexible	Asphalt manufactured at temperatures between 0 and 40 °C; material heating unnecessary	Increased cost-effectiveness; lower energy consumption; decreased environmental degradation; and ease of availability	Inferior performance; lower early life strength; higher voids; and higher moisture susceptibility	[85,91]
Bio-Binders	Flexible	Asphalt binder alternatives made from bio-oil, which can be produced from a variety of biomass materials, including soybean oil, palm oil, vegetable oil, etc.	Increased environmental sustainability and natural resource conservation; increased crack resistance at low temperatures; can diminish asphalt-related toxic fumes	Decreased high-temperature stability; performance issues regarding aging resistance	[[92], [93], [94], [95], [96], [97], [98], [99], [100], [101], [102], [103]]
Recycled Material Bitumen Enhancement	Flexible	Bitumen can be enhanced with waste materials such as reclaimed rubber products, polymers, catalysts, fillers, fibers, extenders, plastic, waste cooking oil, and palm oil fuel ash.	Increased environmental sustainability and natural resource conservation; decreased costs of waste materials	Unknown risk with nontraditional materials; may require treatment to ensure performance; may pose a technical risk or maintenance liability; dependent upon locally available materials	[17,104,105]
Inverted Pavements	Flexible	In inverted pavement designs, a well-compacted granular aggregate base is placed on top of a cement-treated base, then a thin layer of asphalt surface course is placed over the top.	Cost-effective; allows incorporation of sustainable materials; strong structural support and bearing capacity, prevents reflective cracking and propagation from the bound cemented base into the asphalt surface.	The granular base is a key structural element and may require treatment to ensure performance; requires specialized labor, techniques, equipment, and maintenance	[20,21,[106], [107], [108], [109]]
Interlocking Concrete Block Pavement (ICBP)	Flexible	Pavement made from interlocking concrete blocks and is considered flexible pavement; however, it differs from asphalt as it is temperature-independent	High social acceptance; cost-effective; superior structural performance; air-purifying qualities; use of waste materials; reduced noise emission; lower heat island effect	Higher initial costs; lower construction speeds that could cause long-term traffic restrictions; and manufacturers' low interest in producing new block pavers due to costs	[10,110]
Self-Awareness Pavements – Ex. Carbon-doped conductive concrete, optical fiber sensors, etc.	Smart Pavements	Pavements with real-time monitoring capabilities of road conditions such as traffic events, weather, and emergency facilities.	Eco-friendly; strain-sensing capabilities; temperature-sensing capabilities; pressure-sensing capabilities; economically feasible; improved response time	Requires further development and field-testing; can have high initial costs	[111,[112], [113], [114], [115], [116]]
Self-Healing Asphalt Pavements	Smart Pavements	Many self-healing asphalt technologies try to restore and utilize asphalt's inherent self-healing behavior. Technologies include additives and nanoparticles, in-situ heating, and rejuvenation using encapsulation, hollow fibers, or vascular fibers	Increased pavement lifetime; reduces lifecycle costs; reduces emissions related to maintenance	Many of the technologies need further testing before ready for application	[111,[117], [118], [119], [120], [121], [122], [123], [124], [125], [126], [127], [128], [129], [130]]
Self-Healing Concrete Pavements	Smart Pavements	The leading process of self-healing in concrete pavements is through the introduction of bacteria to create calcium carbonate, which can fill microcracks	Increased pavement lifetime; reduces lifecycle costs; reduces emissions related to maintenance; and some have proven to improve concrete strength, durability, and resistance	Less explored than self-healing in asphalt pavements; slow overall process; unknown biological health effects	[[131], [132], [133], [134], [135]]
Information Interaction Pavements	Smart Pavements	Integrated framework design systems for entire roadways that use smart technology to develop integrated applications of building information modeling platforms and intelligent transport system solutions.	Many socio-economic benefits, including improved safety and increased traffic efficiency; reduced maintenance cost due to early detection of defects; increased accessibility through navigation assistive technologies	Still in exploratory stages and require further development to ensure durability and compatibility with existing systems; can have high initial costs; cybersecurity risks; widespread cultural resistance to change	[136,111,112,[137], [138], [139]]
Energy-Harvesting Pavements	Smart Pavements	Intelligent pavements that can take different forms of energy and convert it into electricity using energy transducer devices.	Socio-economic and environmental benefits including providing clean and sustainable energy from renewable sources	Require further development to ensure durability, skid resistance, and compatibility with existing systems; can have high initial costs	[136,[140], [141], [142], [143], [144], [145], [146], [147], [148], [149]]
Cooling Pavements	Smart Pavements	Modified pavements that remain cooler than traditional pavements by reflecting solar energy, enhancing water evaporation, or through other mechanisms.	Reduced urban heat island effect; reduced stormwater runoff and improved water quality; lowered tire noise; enhanced vehicle safety; improved local comfort; enhanced nighttime visibility; significantly improved pavement life, decreased maintenance costs.	Reflective pavements: glare-related issues and decreased outdoor thermal comfort; Evaporative pavements: increased susceptibility to raveling and water damage, lower solar reflectance, difficulty in maintaining water content during warm months	[[150], [151], [152], [153], [154], [155], [156]]
Recycled Material Unbound Pavements	Unbound Pavements	Some recycled materials that can be used in unbound pavements include crushed concrete, crushed brick, crushed glass, and RAP	Recycled materials can perform similarly to natural materials; enhanced material stiffness; improved environmental sustainability	Recycled material variability; may require treatment to ensure performance; may pose a technical risk or maintenance liability; dependent upon locally available materials	[[157], [158], [159], [160], [161], [162], [163]]
Geosynthetic-Reinforced Unbound Pavements	Unbound Pavements	Geosynthetic reinforcement can improve the mechanical characteristics and performance of unpaved roads.	Enhanced durability and road service life; requires less maintenance; fast construction times; environmentally friendly; improved load distribution	High initial cost; requires specialized labor, techniques, equipment, and maintenance; long term durability concerns	[[164], [165], [166], [167], [168], [169]]
Dynamic Monitoring Systems	Unbound Pavements	Smart systems with real-time monitoring capabilities of road conditions, such as an Unmanned Aerial Vehicle (UAV)-based digital imaging system.	Improved maintenance and monitoring, which can lead to cost savings; enhanced safety	High initial cost; needs further development to accurately monitor road conditions and all distresses	
In-Situ Stabilization	Stabilized Pavements	Stabilizing agents are blended with existing materials to stabilize and improve the mechanical properties of the soil or pavement material.	Low environmental impact; reduced construction time, traffic impacts and, in some cases, costs	Recycled material variability, unlikely to be equivalent to conventional properties, higher water susceptibility	[163,[170], [171], [172]]
Biofuel Co-Products Soil Stabilization	Stabilized Pavements	Lignin-based emulsion improves road stability due to the cementitious nature of lignin, a coproduct of biofuel and paper industries.	Eco-friendly; low-energy; low-cost; can improve the mechanical properties of low-quality soils	Requires further development and field-testing	[20,[173], [174], [175], [176], [177], [178], [179], [180], [181], [182], [183], [184], [185], [186], [187]]

### 3.1 High-volume urban pavement innovations results

#### 3.1.1 Rigid pavement innovations

Rigid concrete pavements are designed to transfer wheel loads to underlying layers [188,189]. In their 2016 article, Mohod & Kadam identify four main categories of rigid pavements, including 1) jointed plain concrete pavement, 2) jointed reinforced concrete pavement, 3) continuous reinforced concrete pavement, and 4) pre-stressed concrete pavement [190]. Rigid pavement systems have many advantages compared to flexible pavement systems, which often make them more suitable for high-volume roads. These advantages include a longer lifespan, decreased lifetime cost due to the higher maintenance needs of flexible pavement, and increased durability under service environmental and traffic conditions [188]; according to a cost and benefit analysis, flexible pavement incurs higher maintenance and rehabilitation costs when compared to rigid pavements due to their faster deterioration [191]. Despite these advantages, rigid pavement systems also have some disadvantages, including long-term traffic restrictions due to long curing times and weather restrictions at the time of placement [59].

##### 3.1.1.1 Sustainable materials and mixture technologies

As concrete is the second most used material in the world behind water [192] and the production of cement and concrete is a significant contributor of carbon dioxide (CO_2_) emissions across the globe [2], research on reducing carbon dioxide emissions associated with these industries is becoming increasingly important. Accordingly, there has been extensive effort made to reduce the CO_2_ intensity of cement production, including research from the United Nations Environmental Program, Sustainable Building and Climate Initiative (UNEP-SBCI) [39] and the International Energy Agency with the World Business Council for Sustainable Development [193]. According to the research carried out by the UNEP-SBCI and multi-stakeholder working group [39], two approaches that can deliver considerable reductions in global CO_2_ emissions in the near future are (1) increasing the usage of low-CO_2_ supplementary cementitious materials (SCMs) as partial replacements for Portland cement clinker and (2) utilizing Portland cement clinker more efficiently in mortars and concretes [39].

SCMs, which traditionally include materials such as fly ash [40], blast furnace slag [41], and silica fume, are currently employed as one of the primary tools for reducing carbon dioxide emissions associated with concrete production [42]. Not only are SCMs used to respond to the increasing sustainability concerns of the construction sector [43], but they are also used to increase concrete's resistance to deterioration mechanisms [44], improve its long-term compressive strength, and reduce the associated cost [42]. Other new SCMs include materials such as natural pozzolans, calcined clays, limestone, biomass ash, bottom ash, steel slag, copper slag, other non-ferro slag, bauxite residue, and waste glass [45]. However, while there are many studies on new sources of SCMs and their technical potential, some barriers limit their application, such as their reactivity times or their impact on concrete properties; thus, more research is needed to realize the full potential of the new SCMs [42].

In the long-term, another method of reducing CO_2_ emissions related to cement production is to develop alternative low-carbon binders [39]. Replacing ordinary Portland cement in pavements with alternative low-carbon cements could offer potential carbon benefits, as the direct CO_2_ emissions of OPC clinker (which ranges from 0.809 to 0.843 kg_CO2_/kg) is typically higher than that of alternative low-carbon cements [46]. Using their own theoretical model to calculate the CO_2_ emissions of alternative low-carbon cements, Nie et al. found that calcium sulfoaluminate clinker and high-belite calcium sulfoaluminate clinker produce 0.540 kg _CO2_/kg and 0.333 kg _CO2_/kg process-related CO_2_ emissions, respectively [46]. Despite their benefits, there are economic, technical, practical, and cultural barriers to adopting low-carbon cementitious materials into common construction practices, such as pavement design. The cultural barriers may include the perception of high costs of low-carbon materials, insufficient information provided by material producers, and the risk-averse and litigious culture that pervades the industry; these factors alone often create an unwillingness to adopt unfamiliar materials [47]. Moreover, as an emerging technology that does not have centuries of performance data available, there is more uncertainty about long-term durability, which can also hinder their adoption [47].

Additionally, aggregates represent 70–85 % of Portland cement concrete [11], however, the operations used to acquire aggregate materials (i.e. mining, processing, and transportation) cause environmental degradation, release significant amounts of carbon dioxide emissions, and consume considerable amounts of energy [48]. Utilizing recycled and waste materials as aggregates has the potential for environmental benefits, such as reducing waste in landfills and contributing to the circular economy, as well as reducing the dependency upon virgin aggregate materials and thus reducing the extraction of nonrenewable resources. A wide range of renewable and recycled materials have been investigated to this end [1]. Recycled materials used to replace aggregates include reclaimed asphalt pavement (RAP), recycled concrete aggregate (RCA), recycled asphalt shingles, steel furnace slag, waste foundry sand, waste glass, crushed brick, other construction and demolition waste aggregates [20], and more.

Often, the performance of pavements with recycled materials are similar or even improved compared to conventional pavements. For example, utilizing RAP as an aggregate in pavements offers benefits such as improved rutting resistance; using even 20 % RAP can improve bituminous mixture properties and overall performance [20,49]. When compared to conventional concrete mixes, RCA concrete, with up to 50 % recycled aggregate, generally displays similar or equivalent mechanical properties in all aspects [48]. Additionally, RCA can be used as an alternate aggregate material in both asphalt and concrete mixtures, but when used in the base or subbase layers, it can increase the overall modulus and stiffness of the pavement [20,50]. Recycled asphalt shingles are limited to use as fine aggregate fractions in asphalt mixtures [21]; however, this material is relatively experimental and needs further field testing [20]. Steel furnace slag can be used as an aggregate material in both asphalt and concrete mixtures, improving skid resistance, moisture resistance, and rutting resistance in asphalt mixtures and producing similar properties in concrete mixtures to conventional concrete mixtures [20,51,52]. Waste foundry sand can partially replace fine aggregate in asphalt and concrete mixtures and has been found to positively affect the mechanical properties of concrete mixtures [21,53,54]. Waste glass can also partially replace aggregate in asphalt and concrete mixtures and can improve pavement strength, durability, structural performance, and aesthetics [55]. Finally, crushed brick can be used as a partial replacement in base and subbase layers [20]; however, to perform appropriately and enhance its durability, it must be blended with other durable recycled aggregates [56].

Although some recycled materials, such as RAP, have proven to produce similar or even better-quality results than virgin materials [57], the recycled material must be used carefully in pavements so as not to decrease the overall pavement quality. Many countries regulate the quantity of recycled material tolerated in pavement mixes to safeguard the quality of the pavement. For example, in recycled asphalt mixes, RAP content is limited to 15–20 % in some countries [58]. Furthermore, waste materials are often treated or improved to ensure they meet performance requirements [12]. As Jamshidi & White point out, “The decision to use waste materials in a pavement is a balance between technical risk, maintenance liability, available materials, environmental emissions and capital cost” [12].

##### 3.1.1.2 Precast concrete pavement (PCP) systems

In their article, Novak et al. review the most utilized precast concrete pavement systems used to date of publication, including the hexagonal-shaped panel system and precast concrete pavement system developed by the Soviet Union, the Fort Miller Super Slab system, the Michigan system, the Uretek Stitch system, and the Kwik system [59]. Precast concrete pavement systems are precast concrete panels manufactured and cured at an external location. They are then brought to the construction site, where they are installed and maneuvered into place on prepared base layers. Precast concrete pavement systems have gained much attention throughout recent years since they are not as susceptible to the main disadvantages of traditional rigid pavement systems. For example, precast concrete pavement systems have minimal weather restrictions when placing and require less time to place; thus, they should not cause as many long-term traffic restrictions [59]. Additionally, precast concrete pavement systems can produce better quality concrete as the curing conditions can be better controlled. Nevertheless, precast concrete pavement systems have drawbacks, including a much higher initial cost, load transfer issues created between the panels and existing pavement, and the need for careful leveling to avoid bumps formed between panels [59,60].

##### 3.1.1.3 Ultra-high performance concrete (UHPC) overlays

Concrete overlays are applied on pavements to optimize and extend the lifespan of an existing pavement and can be placed using conventional concrete pavement practices [61]. Ultra-high performance concrete (UHPC) or ultra-high performance fiber reinforced concrete (UHPFRC) consists of concrete with a dense granular matrix, also known as DSP [62], that is fiber-reinforced [63]. UHPC exhibits ultra-enhanced durability and mechanical properties, such as an ultra-high compressive strength [64], extremely high impermeability, negligible drying shrinkage if properly cured, excellent post-cracking tensile capacity, and high early strength, which could reduce traffic closure time [61]. Despite these many benefits, UHPC is typically associated with reduced environmental sustainability and increased cost due to its high usage of Portland cement and silica fume [65]. To make UHPC more eco-friendly and economical, many alternative mix designs have been developed, for example, utilizing micro and nano-sized SCMs to partially replace Portland cement in UHPC [61,63,[66], [67], [68], [69]]. Moreover, while the CO_2_ burden of UHPC is ∼73 % higher than traditional concrete on a per ton basis, CO_2_ emissions can be reduced by 16 % when UHPC is used since significantly less UHPC (about half that of the ordinary Portland cement concrete) is required to construct the same piece of infrastructure [70].

##### 3.1.1.4 Self-compacting concrete

Self-compacting concrete (SCC) is a high strength and high performance concrete that does not necessitate vibration to achieve compaction [71], and is thus considered an energy-efficient material [72]. To achieve a dense state without vibration, SCC mixtures must be able to flow and compact under their own weight. To achieve this, they must have a lowered water-cement ratio and contain more mortar, corresponding to a much higher sand content and less coarse aggregate [73]. One challenge for SCC mixtures is to achieve the required flow without the mix segregating. In other words, the solids must stay well dispersed within the fluid [73]. Although SCC has traditionally been used mostly in the construction of buildings, bridges, and tunnels due to its superior durability characteristics [74], its usage in rigid concrete pavements is being investigated due to its demonstrated material advantages and the potential positive effects it could have [71]. Recently, with the aim of making SCC a more environmentally friendly material, a number of research projects have investigated the viability of incorporating recycled materials in its production [72,[75], [76], [77], [78], [79], [80], [81], [82]]. Based on their extensive literature review, Santos et al. conclude that the use of recycled aggregates to produce SCC “is justified and technically viable,” however, precautions must be taken to ensure the recycled aggregate concretes meet required performance characteristics [82]. Additionally, using recycled aggregate tends to reduce the working performance of self-compacting concrete due to its high water absorption and particle angularity, both of which reduce flowability. The recycled aggregate type, size, and substitution rate are important indexes for satisfying the working performance, mechanical properties, and durability requirements of self-compacting concrete; thus, it is necessary to develop specific standards for use of recycled aggregate in self-compacting concrete [83]. Despite the benefits of SCC, it is susceptible to numerous forms of cracking and other structural defects, limiting its use for rigid pavement applications [71,84].

#### 3.1.2 Flexible pavement innovations

Unlike rigid pavements, flexible pavements do not rely on flexural strength to transfer loads. Flexible pavements rely on grain-to-grain contact between aggregates within underlying layers to transfer loads [188]. In their 2016 article, Mohod & Kadam identify three main categories of flexible pavements, including 1) conventional layered flexible pavement, 2) full-depth asphalt pavement, and 3) contained rock asphalt mat [190]. Flexible pavement systems have some critical disadvantages compared to rigid pavements, including increased maintenance requirements and costs, shorter lifespan, and degradation from extreme weather conditions and excessive loading [188]. Furthermore, when compared to rigid pavements, flexible pavements have increased fuel consumption and decreased nighttime visibility [188]. However, flexible pavements are also more economical for lower volume roads, have a lower initial cost, require less repair time, and produce less traffic noise than rigid pavements [188].

##### 3.1.2.1 Sustainable materials and mixture technologies

Asphalt is a vital part of flexible pavement design. However, it is also detrimental to the environment and human health in many ways, including through its smoke emission [[194], [195], [196]] and its utilization of nonrenewable resources [58]. Asphalt, also known as bitumen, is a form of petroleum, a nonrenewable resource. Hot-mix asphalt concrete (HMA), the most widely used asphalt mix, consists of bitumen and mineral aggregates mixed at high temperatures, between 150 °C and 170 °C, which requires high energy use and results in the production of greenhouse gases [197]. Although HMA has advantages such as superior performance and lower initial cost [[85], [198], [199], [200]], its main disadvantage is its greenhouse gas emissions. In an effort to make asphalt more environmentally and economically friendly, multiple material and technological innovations have been made in the industry, including sulfur extended asphalt, asphalt bio-binders, warm-mix asphalt (WMA), foamed asphalt, rubberized asphalt, polymer-modified asphalt, and cold asphalt emulsion mixtures [20].

Some innovations have been more promising in terms of environmental sustainability than others. For example, WMA technology has substantial benefits compared to traditional HMA. WMA, which is produced and placed at temperatures between 100 and 140 °C, requires lower energy use and thus reduces the carbon emissions associated with the manufacturing processes. Additionally, researchers have observed improved health and safety conditions of personnel and workers working with WMA [86,87]. WMA technologies also offer an extended paving window, fewer restrictions in poor air quality areas, and some improvement in physical and mechanical properties and durability, such as improved workability and compaction efficiency [3,88]. The WMA technologies also allow higher proportions of recycled materials in their mix designs [86]. These recycled materials include reclaimed asphalt pavement (RAP), Recycled Asphalt Shingles (RAS), construction and demolition waste (such as tiles and bricks), and industry by-products (for example, copper or steel slags) [89]. Including RAP material in WMA mixes can enhance WMA performance (e.g., advanced mechanical properties (strength and modulus), rut resistance, moisture damage resistance, fatigue cracking resistance, and low temperature cracking resistance), and decrease the usage of virgin materials, since WMA-RAP mixes can utilize a higher RAP content [90]. Despite the many benefits, WMA still has some weaknesses, such as increased susceptibility to trapped moisture [88], which can cause premature pavement decay. Currently, there are three different commercially available approaches to produce WMA. These are typically categorized as foamed asphalt technologies, organic additives, and chemical additives. In each case, the goal is to facilitate mixing, compaction, and binder adhesion to aggregates at lower production temperatures than HMA [90]. WMA also includes half warm mix asphalt (HWMA), which has a maximum manufacturing temperature less than 100 °C [85].

The third asphalt mix technology is cold mix asphalt (CMA), which is manufactured at temperatures between 0 and 40 °C and does not require any preheating of material [85]. Although CMA has many advantages over HMA, including its cost-effectiveness, lower energy consumption, decreased environmental degradation, and availability, its inferior performance, due to its lower early life strength, higher voids, and higher moisture susceptibility, currently limits its use to minor construction and repair works [85]. In an effort to improve the performance of CMA and make it comparable to HMA, multiple studies have been carried out on the modification of CMA through the incorporation of active fillers, chemicals, fibers, and different waste materials. While some conclude that implementation of nanomaterials and fibers seem to be promising for CMA design, additional testing is needed to evaluate the robustness of the solution by determining how mix design parameters and placement techniques affect CMA properties like stiffness, rutting, and more [91].

Although bitumen is a waste product of refining operations, it is utilized in multiple applications, including pavements; thus, although it is a byproduct, it is not an unwanted one. However, to meet sustainable development requirements and resolve the depletion of petroleum resources, the asphalt pavement industry is exploring asphalt binder alternatives made from non-petroleum-based renewable sources [92], including bio-binders [[93], [94], [95], [96], [97]]. Bio-binders are made from bio-oil, which can be produced from a variety of biomass materials, including soybean oil, palm oil, vegetable oil, microalgae, engine oil residue, grape residues, swine waste, and more [98]. Bio-binders are used to replace or modify petroleum asphalt, creating bio-asphalt [99]. Bio-asphalt can generally be manufactured in three ways: (1) the bio-binder entirely replaces petroleum asphalt (100 % replacement rate); (2) the bio-binder is used to modify petroleum asphalt (less than 10 % replacement rate); or (3) the bio-binder is used as diluent to blend petroleum asphalt (25%–75 % replacement rate) [99,100]. Bio-binders' effects on asphalt mixture properties largely depend on the bio-binder and the percentage used, as well as the application. Compared to traditional petroleum asphalt, bio-based asphalt mixtures have increased crack resistance at low temperatures, but also have decreased high-temperature stability and generally have performance issues regarding aging resistance [99,101]. A team from Arizona State University recently developed a low-carbon, bio-based sustainable pavement binder known as AirDuo [102]. AirDuo not only diminishes toxic fumes of asphalt-surfaced areas, enhancing public health and safety, but also promotes resource conservation and waste valorization [103]. One of the biomass-derived additives AirDuo employs is iron-rich biochar, which stems from the thermochemical conversion of waste biomass like algae and manure [102].

Bitumen can also be enhanced with waste materials such as reclaimed rubber products, polymers (natural and synthetic), catalysts, fillers, fibers, and extenders [201], as well as plastic, waste cooking oil, and palm oil fuel ash [104]. Plastic rubber and polymer-modified bitumen have been extensively used for the construction of roads by many industries for a long time [104,105].

##### 3.1.2.2 Inverted pavements

Inverted pavements were developed in South Africa, where they are still widely used [106]. They are considered an “unconventional” type of flexible asphalt pavement [107] and have considerably low construction and life-cycle costs due to their long lifespans [106]. In inverted pavement design, a well-compacted granular aggregate base is placed on top of a cement-treated base, then a thin layer of asphalt surface course is placed over the top [108,109]. The inverted design provides strong structural support and bearing capacity while also preventing reflective cracking and propagation from the bound cemented base into the asphalt surface [20,21,106]. The high-quality performance of inverted pavement is largely due to utilizing the granular base as a key structural element, thus, the most critical factor in the pavement performance is the quality of the granular base [106]. In South Africa, specifications for aggregates used in unbound bases require the density of the aggregates to be 86–88 % of apparent solid density; in addition, the shape has to meet a sphericity requirement of less than 35 %, and the fines must meet requirements of liquid limit (LL) less than 25 % and a plasticity index (PI) of less than 4 [106].

Additionally, as Plati points out, incorporating “sustainable materials” (i.e. recycled and waste materials) into all layers of inverted pavement is feasible [20]. Thus, inverted pavement is a promising alternative to conventional flexible pavement, due to its high-quality performance, cost-effectiveness, and ability to incorporate sustainable materials [20,106,107].

##### 3.1.2.3 Interlocking concrete block pavement (ICBP) technology

Interlocking concrete block pavement (ICBP) technology is another type of flexible pavement. It differs from asphalt because it is temperature independent [10]. Some other main advantages of the ICBP technology in Japan, are its social acceptance, structural performance, and environmentally friendly characteristics [10].

Due to its use of high-quality materials, ICBP technology achieves sufficient structural performance while also being less sensitive to structural stresses imposed by climate change. Moreover, geofabrics can be utilized to improve the system's subgrade characteristics and loadbearing capacity. Through surveys, it was determined that both able and disabled citizens rated ICBP technology as the best pavement system due to its aesthetic features such as its color, cleanness, convenience, and luminance; low noise emission capabilities; serviceability and rapid maintenance; lower heat island effect; and its positive psychologic effects after disasters such as earthquake and tsunami events [10]. ICBP has been utilized throughout Japan's history and can be found in different historical sites, such as temples and emperor gardens [10]. Japan's culture played a pivotal role in its development, which could also explain its broad social acceptance in Japan [10]. The technology meets environmentally friendly requirements mainly due to its air purifying characteristics and its use of different waste materials, which lowers the extraction rates of nonrenewable resources. It also can be further developed to have energy-harvesting capabilities, such as capturing solar and vibration energy, which would further decrease its environmental impact.

Although it has many benefits, ICBP technology still has some disadvantages, which include higher initial costs, lower construction speeds that could cause long-term traffic restrictions, and manufacturers’ low interest in producing new block pavers due to costs. When analyzing current and future applications of ICBP, it is important to consider that failure and progress depend on multiple factors, such as pavement application, traffic volume, construction quality, and more. Currently, less than 1 % of ICBP in Japan has been used in roads; it is most commonly used in sidewalks, bicycle tracks, and recreational areas [10]. However, using a comparative engineering-economical evaluation and analysis, Ishai concluded that although the upfront construction cost of ICBP is higher than that of the flexible pavement in medium and low-traffic conditions, it is lower than flexible pavement designed for high-traffic conditions [110]. Additionally, the total cost (i.e., the sum of the construction and maintenance costs) of ICBP is always equal to or less than that of flexible pavements and is substantially lower than that of rigid pavement for all traffic categories [110].

#### 3.1.3 Smart and multifunctional pavement

With the continued development of advanced computing technology in the 21st century, such as artificial intelligence, machine learning, and the Internet of Things (IoT), i.e., “embedded devices (things) with Internet connectivity, allowing them to interact with each other, services, and people on a global scale” [202], extensive research is being undertaken by countries around the globe to determine how these technologies can improve traditional pavement systems. Intelligent pavements come in many different forms, including energy-harvesting pavements and systems that can collect and process real-time information about pavement conditions, including data about the stress, strain, and deformation the pavements are subject to Ref. [136]; other intelligent pavements can respond to pavement distress with self-healing capabilities. These technologies’ real-time data collection and monitoring capabilities can significantly improve pavement maintenance routines since road conditions are continuously analyzed. Additionally, combined with machine learning capabilities, the data collection capabilities can capture more accurate and reliable data over time [136]. While the potential benefits of these systems are numerous, most of the technologies are in their early development stages.

**Fig. 3:**
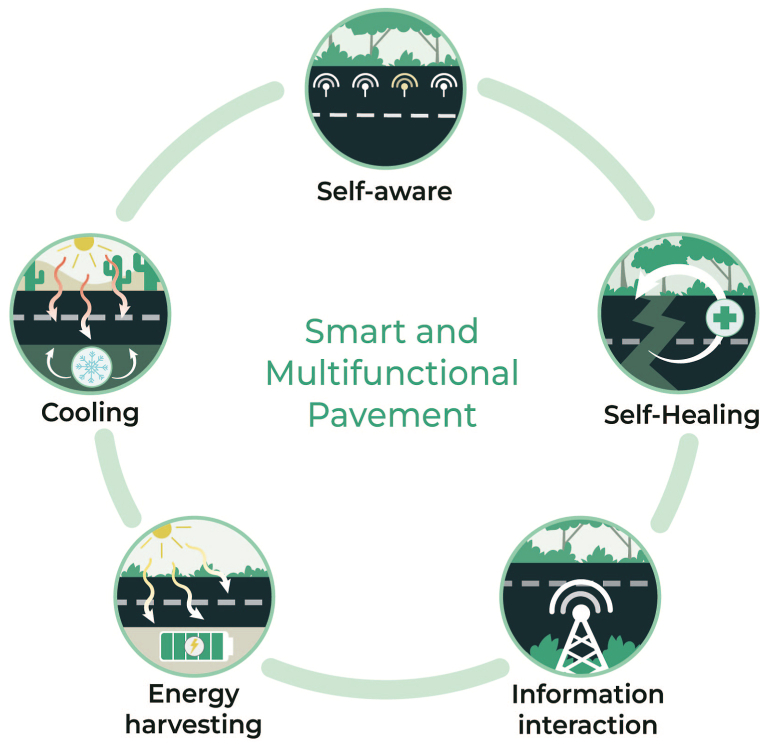
Five categories of smart and multifunctional pavement.

While there are many types of intelligent pavements, smart pavements can generally be categorized into four groups: information interaction, self-awareness, self-adaptation, and energy harvesting [111,203]. This section considers five forms of intelligent and multifunctional pavement technologies and abilities including self-awareness, self-healing, information interaction, energy-harvesting, and self-cooling (Fig. 3). This section is not meant to be an exhaustive review of all smart and multifunctional pavements but rather a broad overview with significant supporting examples.

##### 3.1.3.1 Self-Awareness Pavement

Self-Awareness Pavement refers to pavements with “the ability to monitor the road conditions (even traffic status) automatically and in real-time” [111]. Digitalization in highways can enable real-time monitoring of traffic events, weather conditions, and emergency facilities [112]. At present, it is imperative to explore how intelligent technology can be applied to pavement monitoring systems due to the rising number of vehicles on roadways, which, consequently, causes additional pavement degradation, affecting users’ safety and ride quality [113]. Thus, many researchers are trying to develop real-time pavement monitoring systems to obtain more comprehensive traffic data such as traffic load, traffic volume, and more [114].

In their study, Birgin et al. propose a new composite pavement material doped with carbon microfiber inclusions that possesses weigh-in-motion (WIM) sensing capabilities [115]. The composite material “is doped with carbon microfibers which confer the pavement with piezo-resistive properties producing measurable electrical responses provoked by traffic-induced deformations” [115]. According to their results, the composite material can localize, quantify, and differentiate between applied loads; thus, it can be helpful in condition-based maintenance decisions by providing daily road-usage data and data on extraordinary loading events [115]. The composite pavement material is field-test ready, eco-friendly, has strain-sensing capabilities, demonstrates a quick response time, and is economically feasible. In a follow-up study, Birgin et al. conducted a field investigation to assess a sample of their smart composite pavement with 1 wt% of CMF inclusions [116]. This proposed system is designed to be significantly more low-cost when compared to other WIM sensing technologies, with the sensing material cost comparable to common asphalt materials and the DAQ system (data acquisition system) cost amounting to 50 USD at the prototyping level [116]. Overall, Birgin et al. concluded that the proposed composite self-sensing material is effective at conducting WIM sensing and monitoring traffic loads of different magnitudes; hence, it is ready for field applications and further tests on operating roads [116].

In addition to carbon-doped conductive concrete, many other sensors, like optical fiber sensors (commonly made from Silica fiber and polymer fiber), can measure the strain, temperature, and pressure information of pavement in real-time [114]. Since the early 21st century, optical fiber sensors have been extensively studied and used to monitor the serviceability of pavements [114]. Commonly used optical fiber sensing technologies include Fiber Bragg Grating, Long Period Grating, Optical Time-Domain Reflectometry, Brillouin Optical Time Domain Reflectometry, Brillouin Optical Time Domain Analysis, and Optical Frequency-Domain Reflectometry [114].

##### 3.1.3.2 Self-healing pavements

Self-healing materials is a relatively new field of research in material technology science [117]. The most explored field of study regarding self-healing materials and pavements is the field of asphalt pavements [111]; however, some research has also been conducted on self-healing concrete pavements. Self-healing technology could be revolutionary in road construction, maintenance, and operation, offering extensive potential economic and environmental benefits.

##### 3.1.3.3 Self-healing asphalt pavements

The healing properties of asphalt have been explored since the 1960s [118,119]. Due to asphalt binders' viscoelastic behavior, asphalt possesses an inherent ability to self-heal. Deformation in the asphalt's microstructure, such as cracks and other defects, can be filled through a molecular diffusion process [120]. However, this behavior diminishes over time due to oxidative aging [120]. The asphalt binder is made up of asphaltenes (solid) and maltenes (liquid). During the oxidative aging process, the asphaltenes increase while the maltenes decrease, leading to increased rigidity and deformations [121]. Additionally, the viscoelastic behavior of asphalt is temperature dependent; better healing occurs at increased temperatures [117,122]. Many self-healing technologies applied to asphalt pavements try to restore and utilize their inherent self-healing behavior. In 2015, Tabakovic & Schlangen identified three leading self-healing technologies available for asphalt pavement design: nanoparticles, induction heating, and rejuvenation [111,117]. Since then, other technologies have developed to assist in self-healing asphalt, such as microwave heating or incorporating additives other than nanoparticles.

Nanoparticle technology is one example of a self-healing technology applied to asphalt pavements [118,123,124]. Nanoclay and nanorubber are two examples of nanoparticles that can improve the mechanical and physical properties of asphalt and its ability to self-heal [117,124]. Time and temperature could, however, negatively affect the healing capabilities of the nanomaterials. For example, at high temperatures, some nanomaterials, such as nanorubber, could separate from the asphalt binder [117]. However, nanoparticles are just one type of additive that can improve the self-healing capabilities of asphalt pavement; others include ionomers, supramolecular polymers, shape memory polymers, and some conventional polymer additives such as crumb rubber [118]. Table 2 summarizes how these various additives improve self-healing properties in asphalt.

**Table 2:** Additional additives used to promote intrinsic self-healing of asphalt. Information taken from Anupam et al. [118].

Additive	Mechanism of Self-healing	Explanation
Nanomaterials	Nanomaterial Modification	Driven by surface energy, nanoparticles move toward the tip of a crack to prevent it from growing and heal it.
Ionomers	Reversible Cross-linking	Ions containing polymers create chains within the asphalt. When a crack forms, intermolecular forces push the opposing sides of the crack together to heal the chain; thus, the crack is sealed.
Supramolecular Polymers	Reversible Cross-linking	Monomer chains break upon the formation of a crack, renewing hydrogen bonds to repair the crack.
Shape Memory Polymers	Shape Memory Effect	The formation of a crack in asphalt containing shape memory polymers changes the permanent shape of the polymers; however, regaining the permanent shape heals the crack.
Conventional Polymers – e.g. Crumb Rubber	Polymer Modification	Polymers modify asphalt binder properties by changing their microstructures. The rubbery supporting network of the polymer modified binder can enhance elastic response which can improve instantaneous healing. Additionally, the binder mixture can promote cohesive healing.

Induction heating and microwave heating are also methods used to activate the self-healing properties of asphalt [125]. The mechanism through which induction heating can take place is through the incorporation of electrically conductive fillers and fibers in asphalt mix, such as aluminum, carbon, graphite, or steel wool fibers, nanotubes, or particles [118]. The fibers are then heated with induction heating, and the diffusion of the asphalt binder is activated [117]. Due to this activation, the asphalt has the ability to move and can thus seal cracks through capillary flow [126]. Contrary to induction heating, microwave heating does not require additives, therefore decreasing the cost and effort associated with the technology [118]. In their study, Norambuena-Contreras & Garcia concluded that microwave heating is better at increasing the temperature of the asphalt binder and, thus, is better at healing asphalt [125]. Despite this, microwave heating degrades the bitumen and increases the porosity of the asphalt mix with every healing cycle [125].

**Fig. 4:**
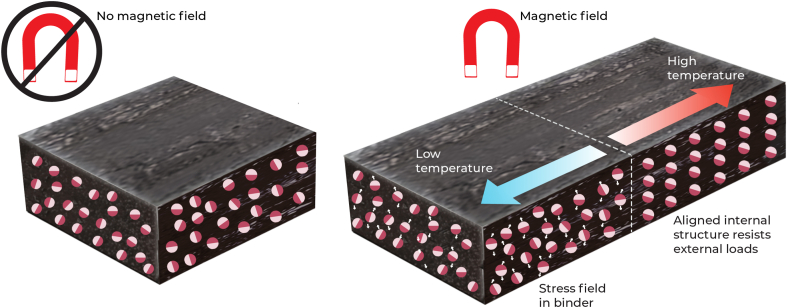
Mechanomutable asphalt binders.

Mechanomutable asphalt binders are a new pavement material that has a bituminous matrix with magnetically susceptible materials [127]. As shown in Fig. 4, using the effects of magnetic fields, the temperature of the binder can be manipulated as the magnetically and electrically responsive materials in the asphalt mixture are attracted to the static magnetic fields created by induction and microwave heating [127,128]. This thus induces the flow of the binder which can repair cracks [126,128].

Finally, rejuvenation is a popular method used to accomplish self-healing in asphalt. Rejuvenators are defined as “an engineered cationic emulsion containing maltenes” ([117], p. 14). They can heal asphalt pavement by restoring the asphaltenes/maltenes ratio in aged bitumen, thus recovering the original properties of the asphalt binder [121]. The addition of rejuvenating agents is common for high RAP content asphalt. The residual bituminous binder in RAP is heavily oxidized and brittle, thus, rejuvenators are added to bring it back to a condition similar to virgin binder. These are typically oils and could even be made of the maltene fraction of bitumen [129]. Rejuvenators can be incorporated into asphalt mix in different ways, including encapsulation, hollow fibers, and vascular fibers [118]. Encapsulated rejuvenators are the most popular form of introducing rejuvenators into the asphalt mix. In this method, the rejuvenator is encased in a shell which is then added to the asphalt mix, if a crack appears in the mix at the site of the encapsulated rejuvenator, the shell breaks and the rejuvenator is released into the mix [118]. A downside to this approach is that it is limited to one-time use.

Overall, using a life cycle analysis (LCA) framework, it was observed that self-healing asphalt pavements increase the lifetime of pavement by 10 % (from 20 years to 22 years) compared with asphalt pavements without any self-healing capacity [117]. Furthermore, compared with traditional roads, the emissions in the life cycle of self-healing pavement can be reduced by about 16 %, and the costs can be reduced by about 32 % [130]. According to a recent review of self-healing pavement technologies, it was found that (1) indoor research proves that the potential of microwave induction heating technology is higher than that of electromagnetic induction heating technology; however, microwave induction heating still causes uneven heating, and (2) the repair potential of the hollow fiber method is higher than that of microcapsule technology, but its material synthesis is more complicated [130]. Moreover, a prospective way to transition from experimental testing to practical application is to explore the synergies between different existing self-healing technologies. For example, Photorepair technology, a little-studied technology that repairs micro-cracks by using light stimulation to change the chemical bonds inside the material, is currently very limited to surface layer repair; however, it can potentially cooperate with other technologies [130].

##### 3.1.3.4 Self-healing concrete pavement

Although less explored than self-healing in asphalt pavements, there have been strides in producing self-healing in concrete pavements. The leading process of self-healing in concrete pavements is through the introduction of bacteria [131]. When combined with a calcium nutrient source such as concrete, *Bacillus Pasteurii*, an enzyme in Ureolytic bacteria, can produce calcium carbonate, which can be used to fill microcracks in concrete [132]. The encapsulation of bacteria may be achieved through various techniques that demonstrate differing healing ratios, i.e., the ratio of the healed crack region to zones of early cracking [132]. For example, polymeric microcapsules based on melamine used for the encapsulation of spores have demonstrated a healing ratio between 48 % and 80 % [132]. Additionally, encapsulation of bacteria with hydrogel bioreagents has achieved healing between about 40 % and 90 % [132]. While multiple methods exist to introduce the bacteria into concrete, the encapsulation incorporation technique produces the best results [132]. This self-healing mechanism is environmentally friendly and has been proven to improve concrete strength, durability, and resistance [132,133]. However, the overall process is slow, and the biological health effects of the bacteria are unknown [134]. The encapsulation technique also controls many properties of the concrete, such as the “behavior of crack propagation, kinetics of healing agent in discrete crack surfaces, [and the] effect of inserted capsules on the mechanical properties of self-healed cementitious materials” [133]. Although bacterial concrete is the most popular mechanism for self-healing concrete, other mechanisms of self-healing in cementitious materials include autogenous self-healing, self-healing based on mineral admixtures, and self-healing based on adhesive agents [135]. However, as stated by Huang et al. “not any particular method of self-healing is the best, but one can be the most suitable for a particular situation” ([135], p. 499).

More recently, Rosewitz et al. have been developing a self-healing mechanism utilizing the Carbonic Anhydrase (CA) enzyme [134]. Within this mechanism, “CA catalyzes the reaction between Ca^2+^ ions and atmospheric CO_2_ to create calcium carbonate crystals with similar thermomechanical properties as the cementitious matrix” [134]. The CA enzyme can be applied to the damaged concrete pavement during maintenance or be incorporated into the cement-paste mix to enable self-healing properties. This mechanism is particularly exciting as it is significantly faster than bacterial concrete, is environmentally friendly due to its consumption of CO_2_, and is inexpensive and biologically safe [134]. While this self-healing mechanism has exciting potential, it is still in the laboratory phase and needs further development and exploration before it can be used in the field.

##### 3.1.3.5 Information interaction pavements

Advancements in technology have reshaped how we can judge pavement systems’ efficiency, safety, productivity, and reliability. One of the biggest challenges in the sector currently includes the efficient management of large-scale roadways [137]. If not properly maintained, deformations can occur in roadways, decreasing the quality of life for citizens and potentially leading to accidents.

Much research is being undertaken on utilizing smart technology to develop integrated framework design systems for entire road systems instead of singular-purpose innovative technology used in lone roadways [111]. Intelligent technology can organize data from sensor networks and thus encourage innovation, automation, connectivity, cooperation, proactivity, safety, and cost savings [111]. Utilizing intelligent technology in roadway systems, with integrated applications of building information modeling platforms and intelligent transport system solutions, is promising for both construction and management practices [111]. For example, an innovative technology that can be utilized in construction practices is the “intelligent compaction technology of asphalt pavement”, which employs a GPS positioning system and embedded vibration characteristic testing equipment to collect real-time data about the machine and road surface [136]. While this can be beneficial for monitoring the quality of the compaction process, the technology is still at an exploratory level and requires further development [136].

Integrating distinct modules, such as communication systems, is essential to continuously communicate data from heterogeneous sources, such as vehicles, roads, and roadside sensors [112]. Vehicle-to-Infrastructure communication is bi-directional wireless communication between vehicles and road infrastructure [112], which aims to support vehicular safety applications, such as collision avoidance, collision detection, and more, as well as mobility applications, such as traffic notification, efficient fuel consumption, smart parking, electronic toll collection, and more [138,139]. Overall, if used correctly, this could provide numerous great socio-economic benefits, such as improved safety, reduced road accidents, and increased traffic efficiency [138,139].

In their article, Dong et al. develop and propose a pavement management system (PMS) that utilizes advanced technologies, such as IoT and big data, to provide an overall management structure for road maintenance [137]. The PMS comprises three sections: (1) pavement detection and 3D modeling, (2) data analysis and decision support, and (3) automated and intelligent solution development and suggestion [137]. The authors state the PMS has the following advantages compared to traditional management systems, “automated high-precision road distress detection, 3D distresses quantification, road distress information extraction based on algorithm, collaboration with other urban systems, and distress development trend estimation” [137].

##### 3.1.3.6 Energy-harvesting pavements

Energy-harvesting pavements are a form of intelligent pavements that take different forms of energy and convert it into electricity using energy transducer devices [136]. This topic has been investigated recently as a potential solution for increasing global energy demands. For example, these pavements can convert the mechanical energy generated by vehicle impact into electricity using piezoelectric, electrostatic, or electromagnetic techniques [140]. These pavements can also convert solar radiation to electricity using solar-thermal techniques, including thermo-electric and pyroelectric generation methods or solar-electrical techniques, through the use of solar photovoltaic technology [140]. Additional energy sources that intelligent pavements can harvest are geothermal [141], wind, and water [140].

**Fig. 5:**
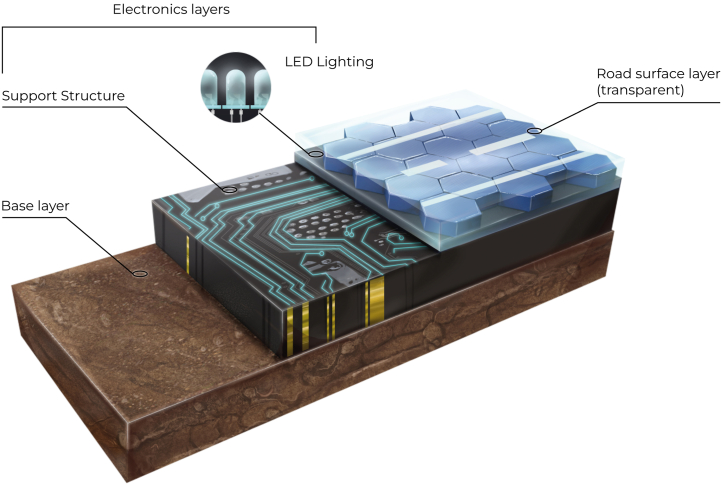
Solar road cross-section.

While these technologies have strong potential to meet the world's increasing energy demands in the future, the technologies need further development before they can be implemented. For example, the solar panel road [142], although reasonably developed in their harvesting efficiency, still poses challenges when harnessed in roadways, such as road operation and skid resistance [143]. Solar roads typically consist of three layers, including, from bottom to top, a base layer, an electronics layer, and the transparent road surface layer (Fig. 5) [144]. The base layer can contain recycled materials; however, it must be weatherproof, as its primary purpose is to support the other two layers and distribute the power collected from the electronics layer [145]. The electronics layer houses the solar cell array and consists of two parts – the LED layer and the support structure. The LED layer can be used to make road markings, such as lanes, and communicate with drivers, for example by projecting signs to signal upcoming road conditions or provide emergency warnings [146]. The transparent surface layer is meant for vehicles to drive on; however, this layer poses many challenges as it must be sufficiently transparent to guarantee the efficient collection of solar radiation by the electronics layer while simultaneously being weatherproof, skid-resistant, and durable enough to withstand traffic conditions. The transparent surface layer must also provide sufficient structural performance (e.g., strength, stiffness, stability, durability, fatigue resistance, and impact resistance). The most common materials used for the transparent surface layer are inorganic materials, such as glass or toughened glass, and high molecular polymers, such as polycarbonates, Plexiglass, or resin [146]. However, these materials are limited in their ability to produce a balanced design between skid resistance and light transmittance [146].

Solar roads also have a high initial cost due to their requirement of inverters and storage batteries to guarantee a constant electricity supply [140]. Solar-electrical techniques not only incorporate use of photovoltaic technology in the physical pavement design, but this type of energy harvesting can also include roadside photovoltaic installation, including noise barriers [147], or above-road solar installation. Highway Right of Way areas are potential areas that could be used for solar energy generation due to their prime physical and topographical characteristics and extensive usage history in Europe [148].

Alternatively, there are also systems that extract solar energy from asphalt concrete without impacting the structure's performance in its primary functions [149]. For example, García & Partl formed a solar turbine by creating artificial porosity in asphalt concrete, which, when connected to an updraft or to a downdraft chimney, can create air flow due to differences in temperature [149]. This solar turbine can then be used to harness energy and manipulate the pavement temperature, which could be used to decrease the urban heat island effect. However, to maximize the air flow, it is imperative to reduce the energy loss through the chimney [149].

Overall, if further developed, these technologies could be a way to produce clean and sustainable energy from renewable sources. The positive effects of this could be seen at multiple levels, including decreasing society's dependence on fossil-fuel energy sources, which will benefit the environment and society. Additionally, researchers believe these pavements could bring economic benefits; however, the economic efficiency of these technologies, when produced at a broad scale, needs to be further evaluated [141].

##### 3.1.3.7 Cooling pavements

Cool pavements are modified to remain cooler than traditional pavements through the reflection of solar energy and the enhancement of water evaporation, or other modifications, including the use of newer approaches such as coatings or grass pavements [150]. Due to paved surfaces both storing excess thermal energy and affecting surrounding air temperature, urban areas possessing more paved surfaces tend to have a relatively higher temperature than surrounding rural areas [[151], [152], [153]]. This phenomenon, known as the urban heat island effect [154], can result in decreased air quality, increased risk of heat-related illness or death, increased energy consumption and greenhouse gases, impaired water quality, and more [155]. Additionally, urban heat islands disproportionately affect low-income communities with higher populations of people of color, who are more likely to live in historically redlined neighborhoods with less vegetative cover [156]. Although cool pavements are still at an early stage of development, not only do they have the potential to mitigate the detrimental effects and inequity of urban heat islands, but they can also significantly improve pavement life [152]. Other benefits of cool pavements include reduced stormwater runoff and improved water quality, lower tire noise, enhanced vehicle safety, improved local comfort, and enhanced nighttime visibility [150].

There are three main types of cool pavements: 1) reflective pavements, which either utilize alternative pavement materials, such as fly ash, slag, or heat-reflective coated aggregates, or utilize pavement coatings, such as an Infra-Red reflective colored coating, a thermochromic coating, or other highly reflective coatings; 2) evaporative pavements, which include porous pavements, pervious pavements, permeable pavements, or water-retaining pavements; and 3) heat storage-modified pavements, such as energy-harvesting pavements, high-conductive pavements, or PCM-incorporated pavements [152]. Despite the superior effectiveness of heat storage-modified pavement compared to reflective and evaporative pavements, reflective and evaporative pavements are more commonly used due to their reduced initial and operating costs and their more straightforward construction procedures [152]. Nevertheless, reflective and evaporative pavements have their drawbacks. The main limitations of reflective pavements include glare-related issues and a reduction in outdoor thermal comfort due to reflected radiations, while the main limitations of evaporative pavements include their susceptibility to raveling and water damage due to their high air void content, lower solar reflectance increasing their absorbed solar radiation, and difficulty maintaining water content during summer, which leads to elevated pavement temperatures [152]. As evaporative pavements rely on evaporative cooling, the pavement water content is crucial; thus, they work best in rainy and humid environments [152].

### 3.2 Low-volume rural pavement innovations results

In addition to the high-volume urban pavement innovations discussed above, much innovation has been made in low-volume, rural pavements, such as unbound granular and stabilized pavements. While there is an argument as to whether unbound granular pavements are totally outside of flexible, rigid, and composite pavements or whether they can be slotted into each of the categories, we have chosen to discuss these pavements in their own section. These technologies provide significant sustainability and climate resilience benefits, and offer strong potential for developing countries or countries with very vast but sparse networks. Although they are designed to support lower volume traffic, unpaved roads are imperative for the growth of rural economies and social development in low- and middle-income countries [17,204].

#### 3.2.1 Unbound granular pavements

Many regions of the world have large sections of unsealed roads. For example, according to the African Development Bank, unpaved roads currently make up 53 % of roads in Africa; less than half of Africa's rural population has access to an all-season road [205]. Additionally, approximately 65 % of roads in Australia are unsealed [15], and 33 % of the complete road network in the United States is unpaved [206,207]. Compared to sealed roads, unsealed roads can are prone to more environmental degradation [15] and are more susceptible to deterioration from traffic and climatic conditions, thus requiring more regular maintenance [17,208]. Since a key part of multiple United Nations Sustainable Development Goals is rural accessibility, effective maintenance of unsealed roads is crucial in low- and middle-income countries to realize growth and economic and social development [17,204]. Regular maintenance of unbound granular pavements includes, for example, the cleaning of roads, cleaning and maintenance of drainage systems, removal of storm damage, mowing of grass, pruning of shrubs and bushes in the road reserve and drains, etc. [208].

##### 3.2.1.1 Recycled materials in unbound pavements

Many countries worldwide allow recycled aggregates in road construction, particularly in unbound and stabilized pavement applications [157,158]. According to Queensland's Department of Transport and Main Roads (Australia), some recycled materials that can be utilized in unbound pavements include crushed concrete, crushed brick, crushed glass (up to 20 %), and RAP [159]. Multiple studies have investigated the use of construction and demolition waste (C&DW) materials in low-volume unpaved roads [158,160,161]. Not only did these studies find that recycled materials can meet respective requirements and often perform similarly to natural materials, Huber et al. highlight the technical benefits of using C&DW materials instead of natural raw materials for specific applications, such as in unpaved roads [160]. In their study, Huber et al. evaluated the field performance of C&DW materials by comparing the performance of mixed C&DW material (mainly crushed concrete and crushed brick) with two natural reference materials (crushed limestone) in unpaved roads throughout surface application field tests [160]. They found that in the long term, the C&DW materials performed at the minimum similarly, but mostly superior, to the natural materials for certain applications (i.e. in unpaved roads) specifically from a material stiffness perspective [160].

Queensland's Department of Transport and Main Roads (TMR) in Australia is also making considerable progress in this field [157,159,162,163]. In a National Asset Center of Excellence (NACOE, a collaboration between TMR and the Australian Road Research Board) multi-year project entitled *P94: Optimizing the Use of Unbound and Stabilized Recycled Pavement Materials in Queensland*, researchers explored the increased use of recycled materials in unbound pavements, specifically for the Queensland Department of TMR [157]. The overall objective of the P94 project was, “to identify how the use of recycled materials can be optimized on TMR projects to achieve cost, sustainability, and long-term performance benefits” [157]. Overall, the project spanned three years, and the primary outcomes were a literature review discussing existing practices of using recycled materials in road pavements in Australia, laboratory evaluations of recycled materials from Queensland, and research dissemination materials [157]. The researchers on this project concluded that recycled materials show similar performance to natural/quarried materials; thus, they updated relevant Transport and Main Road specifications outlining the use of recycled materials in roads [157].

##### 3.2.1.2 Geosynthetic-reinforced unpaved roads

Although geosynthetic materials can be used to reinforce both paved and unpaved roads, this section will focus on geosynthetic reinforcement of unpaved roads. Geosynthetic-reinforcement can improve the mechanical characteristics and performance of unpaved roads and has been used since the 1970s [164]. Although traditional alternatives to geosynthetic-reinforcement, including the substitution of poor foundation soil or the use of greater fill heights, have been used in the past, when compared to the traditional alternatives, the use of geosynthetics is easier, quicker, and better for the environment [165]. Additionally, research shows performance improvement in geosynthetic-reinforced unpaved roads, including enhanced durability and road service life, as well as other advantages, including decreased cost due to reductions in the thickness of the base course [166].

In unpaved roads, two types of geosynthetics are typically used: geogrids and geotextiles [164]. Using moving wheel load field tests, M. Singh et al. confirmed that (1) unreinforced road sections exhibited significantly more surface deformation than reinforced road sections under the same number of vehicle passes and (2) the geotextile-reinforced section performed better than the geogrid-reinforced section [166]. The performance of the test sections (including geotextile-reinforced, geogrid-reinforced, and unreinforced) were analyzed based on the rut depth measurements resulting from the moving wheel load tests [166]. Despite the improved performance of reinforced roads, disadvantages include the associated high initial cost. However, as demonstrated by a cost analysis performed by Palmeira & Antunes, although reinforced unpaved roads have a greater initial cost, reinforced roads require less maintenance and thus produce important savings in the overall cost of the road [165]. Additionally, geocells are another geosynthetic material used for soil stabilization. They are three-dimensional and made of geosynthetics such as geotextiles and/or geogrids [167]. Although these technologies improve load distribution in unreinforced pavement [168], the cost-effectiveness of geocells will vary depending on local context, including factors like traffic, subgrade, material unit costs, etc.; a cost analysis must be carried out to ensure it is an appropriate economic alternative to traditional road base layers [169].

##### 3.2.1.3 Monitoring systems

As stated previously, compared to paved roads, unpaved roads are more susceptible to deterioration from traffic and climatic conditions; accordingly, they require more regular maintenance [17,208]. To ensure proper pavement maintenance, routine pavement monitoring is important for evaluating pavement conditions so that pavement deformations can be identified and resolved to ensure safe and reliable transportation for users [113]. According to previous research, some common deformations that affect unpaved roads are rutting, pulverization, potholes, loose gravel, erosion, and corrugations [113,[209], [210], [211]]. Shtayat et al. point out that there is little research on monitoring systems implemented for unpaved roads [113]. However, three significant case studies implement dynamic monitoring systems in unpaved road systems [[212], [213], [214]]. Monitoring systems for unpaved roads often require manual observation methods, such as the “walk and look” method or the ride comfort rating method [212,213]; however, manual observation is often very time-consuming and does not give reliable data on the deformation severity [113]. In their research, C. Zhang & Elaksher propose an innovative Unmanned Aerial Vehicle (UAV)-based digital imaging system to monitor rural, unpaved roads, which, according to their experiments, provides high accuracy and reliable results [214]. Although their method can produce an accurate 3D model of surface distresses, it can only detect rutting and potholes, thus, not all distresses are monitored [113].

#### 3.2.2 Stabilized pavements

Stabilization refers to “a process by which the intrinsic properties of a pavement material or earthworks materials are altered by the addition of a stabilization binder or granular material to meet performance expectations in its operating, geological and climatic environment” [215]. Stabilization is utilized when sub-grade soils are soft and unsuitable to make a stable base for road construction. Although replacing the poor-quality local natural aggregates or sub-grade soils is a possible solution, this is typically costly, making stabilization the preferred approach [216].

Stabilization techniques vary depending on the binder used. Typically, binders include lime, cement, bitumen (including foamed bitumen or bitumen emulsions), cementitious blends, granular materials, or chemicals [215]. However, less traditional binders can also be used, such as polymers [217,218] and enzymes [219]. Additionally, some recycled materials that can be utilized in stabilization blends include crushed concrete, crushed brick, crushed glass, RAP, fly ash and slag, and in-situ material [159]. To understand more about the performance and the mechanical properties of recycled material blends, Zhalehjoo & Grenfell used a laboratory testing program to investigate how different stabilization blend proportions of recycled material perform in different scenarios. They concluded that foamed bitumen stabilization is a feasible and viable method to improve the engineering properties of recycled material blends, however, the suitability of these blends depend on several factors including recycled material type and source, type of stabilization, fines content and particle size distribution overall, and other physical properties [220]. To determine the most suitable binder or stabilization agent, many factors must be considered such as price, local availability, material characteristics, durability, and local government policy [221].

##### 3.2.2.1 In-situ stabilization

In-situ stabilization refers to “the process of blending existing materials with stabilizing agents … to strengthen and rejuvenate the soil and/or pavement structure without removing the material” [163]. This is done to improve the mechanical properties of the existing soil or pavement material. Some of its benefits include a reduction in environmental degradation (through the reuse of existing materials, reduction of generated waste, and reduction in transportation emissions) as well as significant reductions in construction time, traffic impacts [163], and, in some cases, costs. In-situ stabilization can be done using multiple methods including the cold recycling/mixing process, which is more cost effective than traditional methods and better for the environment [170,171]. However, as cold recycling is carried out in ambient temperature, bitumen emulsion and foamed bitumen are often used as binders, thus resulting in a more gradual binding process [170,172]. Although economically and environmentally beneficial, a drawback of cold in situ recycling is that it utilizes recycled materials that are inherently more variable than virgin mixes. Although the product will have ‘reasonably high strength,’ it is unlikely to be equivalent to conventional HMA, will take time to develop, and its water susceptibility will be higher [170].

##### 3.2.2.2 Biofuel co-products

Moreover, in an effort to reduce soil stabilization costs, as well as contribute to sustainable development, the potential of biofuel co-products (BCPs) in soil stabilization have been explored [20,[173], [174], [175], [176], [177]]. Lignin, a coproduct of biofuel and paper industries [173], is the second most abundant plant polymer on earth [178] and studies have demonstrated that lignin-based BCPs are a promising additive for soil stabilization [[173], [174], [175], [176]]. It is also beneficial for dust suppression by protecting against erosion in desert climates [173]. Due to the cementitious nature of lignin, lignin-based emulsions can be used to improve the stability of roads since the material can occupy interparticle pores and facilitate the bonding of soil particles. For lignin-based stabilized soil, the main parameters contributing to stabilization are the soil, lignin, mixing, curing, and compaction [179]. Lignosulfonates can be purchased in liquid concentrate or dry powder form, but once it is delivered to the application site it must be mixed with water to achieve the desired concentration level prior to application [180]. When used for dust suppression, lignosulfonates can be applied using a sprayed-on or mixed-in method; however, when used for soil stabilization, a deep mixed-in method (typically 4 to 8 in) is required with an application rate based on the desired degree of stabilization [180]. To accomplish this, the soil is first loosened to the desired treatment depth, and then using a tanker or water truck with a spray bar, the lignosulfonate is applied uniformly, often in multiple passes, and mixed with the loose soil [180]. After thoroughly mixing the soil and lignosulfonate, the soil mix is then graded and compacted. Finally, as an option step to reduce surface water infiltration and lignosulfonate leaching, a thin asphalt surface treatment can be placed on top [180].

While lignin is an eco-friendly, low-energy, low-cost soil stabilizer, more research needs to be conducted to further investigate the applicability of lignin for soil stabilization [178]. Although studies have found that using lignin for soil stabilization can improve the mechanical properties of low-quality soils, such as compressive strength, freeze-thaw durability, moisture susceptibility, and shear strength of soil bases [176,178,[181], [182], [183], [184], [185]], using lignin as a soil stabilizer has only been investigated very recently and related research is still quite limited. Many previous studies have been done at a laboratory scale; thus field trials need to be conducted to further understand the effects of using lignin as a stabilizer [186]. In their paper, Zhang et al. indicate important future areas of research regarding lignin, including lignin optimization/modification, dynamic behaviors of stabilized soils, and application in some special soils [187]. Furthermore, the interaction between lignin, soil, and water still needs to be further explored and understood to achieve the best stabilization results [178].

## 4 Conclusion

As the transportation and pavement industries continue to advance, it is essential to remember some crucial elements regarding change and sustainability while moving forward. (1) First, pavements should be designed to be adaptable to changing traffic inputs and environmental conditions and fulfill the requirements of the end-users, including safety, durability, comfort, efficiency, and economic necessities. (2) These factors, i.e., safety, durability, comfort, efficiency, and economics, may be defined and scaled differently by different stakeholders. Stakeholders' wants and needs, especially those of the marginalized and the most directly impacted stakeholders, should be discussed, considered, and designed for when it comes to their roadways. (3) In the pavement industry, sustainable design objectives should aim at “environmental awareness and compliance, simultaneously adapting to economic, budgetary limitations while at the same time also fulfilling the emerging societal needs and demands” ([20], p. 541). Sustainability should not consider only the environment; other aspects must be considered to ensure the pavements are sustainable, including the economy and the people. Therefore, understanding the context of the communities where the pavement is being placed, such as the cultural norms, socioeconomic status, local environment, etc., is essential. Finally, (4) it is possible to quantify environmental, economic, and social sustainability using tools such as LCA, TEA, and *S*-LCA. Innovations in pavement design and pavement installations should always employ mechanisms to ensure sustainability throughout the design. Too often, sustainability is assumed, for example, by simply using renewable materials to substitute aggregates, but not quantified to ensure that new pavements are actually more sustainable than the counterparts that they aim to replace.

This review has highlighted some important innovations in the pavement industry, with a focus on the sustainability of these systems. While the progress made thus far has been significant, there is still much work to be done to implement robust, sustainable, and economical solutions. Many of the technologies discussed are still in exploratory research phases; it will take more time for the technologies and theories to advance before they can be field-tested. Continued innovation in this field necessitates collaboration between different areas, including researchers, practitioners, engineers, stakeholders, and public-private organizations.

## Data availability statement

The data analyzed in this review is available in the referenced materials and will be provided upon request.

## CRediT authorship contribution statement

**Jaime Styer:** Writing – review & editing, Writing – original draft, Visualization, Validation, Methodology, Investigation, Formal analysis, Data curation, Conceptualization. **Lori Tunstall:** Writing – review & editing, Writing – original draft, Visualization, Validation, Supervision, Resources, Project administration, Methodology, Investigation, Funding acquisition, Formal analysis, Data curation, Conceptualization. **Amy Landis:** Writing – review & editing, Writing – original draft, Resources, Methodology, Investigation, Data curation. **James Grenfell:** Writing – review & editing, Writing – original draft, Validation, Supervision, Resources, Project administration, Methodology, Investigation, Formal analysis, Data curation, Conceptualization.

## Declaration of competing interest

The authors declare the following financial interests/personal relationships which may be considered as potential competing interests: Lori Tunstall reports financial support was provided by Center for 10.13039/100008086Global Development. If there are other authors, they declare that they have no known competing financial interests or personal relationships that could have appeared to influence the work reported in this paper.

## References

[1] Salehi S., Arashpour M., Kodikara J., Guppy R. (Sep. 2021). Sustainable pavement construction: a systematic literature review of environmental and economic analysis of recycled materials. J. Clean. Prod..

[2] UNEP, “Building sector emissions hit record high, but low-carbon pandemic recovery can help transform sector – UN report,” UN Environment. Accessed: February. 28, 2022. [Online]. Available: http://www.unep.org/news-and-stories/press-release/building-sector-emissions-hit-record-high-low-carbon-pandemic.

[3] Wang H., Liu X., Apostolidis P., Scarpas T. (Mar. 2018). Review of warm mix rubberized asphalt concrete: towards a sustainable paving technology. J. Clean. Prod..

[4] Ritchie H., Roser M. (Jun. 2020). CO₂ emissions: how much CO₂ does the world emit? Which countries emit the most?. Our World in Data.

[5] Hickel J. (Sep. 2020). Quantifying national responsibility for climate breakdown: an equality-based attribution approach for carbon dioxide emissions in excess of the planetary boundary. Lancet Planet. Health.

[6] CGD and J. Busch, “Climate Change and Development in Three Charts,” Center For Global Development (CGD) | Ideas to Action. Accessed: December. 5, 2023. [Online]. Available: https://www.cgdev.org/blog/climate-change-and-development-three-charts.

[7] Ritchie H. (Dec. 2023). Our World in Data.

[8] Ceschin F. (2014). How the design of socio-technical experiments can enable radical changes for sustainability. Int. J. Des..

[9] Scott J. (1998). Seeing like a State: How Certain Schemes to Improve the Human Condition Have Failed.

[10] Jamshidi A. (Mar. 2019). State-of-the-art of interlocking concrete block pavement technology in Japan as a post-modern pavement. Construct. Build. Mater..

[11] Papagiannakis A.T., Masad E.A. (2017).

[12] Jamshidi A., White G. (Jan. 2020). Evaluation of performance and challenges of use of waste materials in pavement construction: a critical review. Appl. Sci..

[13] Long B., Shatnawi S. (Jan. 2000). Structural evaluation of rigid pavement sections. Road Mater. Pavement Des..

[14] TMR (2015).

[15] Arrb (Oct. 2020). “Unsealed Roads Best Practice Guide: Edition 2,” Australian Road Research Board (ARRB) Group, Port Melbourne, Australia, Best Practice Guides.

[16] Hine J., Sasidharan M., Torbaghan M.E., Burrow M., Usman K. (Jul. 2019). K4D Knowledge, Evidence and Learning for Development.

[17] Workman R., Wong P., Wright A., Wang Z. (Jan. 2023). Prediction of unpaved road conditions using high-resolution optical satellite imagery and machine learning. Rem. Sens..

[18] Crane A., Matten D. (2019). Business Ethics.

[19] J. Elkington, “25 Years Ago I Coined the Phrase ‘Triple Bottom Line.’ Here's Why It's Time to Rethink It.,” Harvard Business Publishing. Accessed: October. 11, 2021. [Online]. Available: https://hbsp.harvard.edu/product/H04E7P-PDF-ENG.

[20] Plati C. (Jun. 2019). Sustainability factors in pavement materials, design, and preservation strategies: a literature review. Construct. Build. Mater..

[21] Van Dam T.J. (Jan. 2015). Towards sustainable pavement systems: a reference document. FHWA-HIF-15-002.

[22] Institute Iser S.E. (Jul. 08, 2023). PCR 2019:14 Construction Products (EN 15804+A2).

[23] EPD (May 18, 2022). PCR 2019:14-C-PCR-001 C-PCR-001 Cement and Building Lime (EN 16908) (2022-05-18).

[24] Strazza C., Del Borghi A., Blengini G.A., Gallo M. (Jul. 2010). Definition of the methodology for a Sector EPD (Environmental Product Declaration): case study of the average Italian cement. Int. J. Life Cycle Assess..

[25] EPD (Jan. 02, 2023). PCR 2019:14-c-PCR-003 c-PCR-003 Concrete and concrete elements (EN 16757) (2023-01-02). https://environdec.com/pcr-library.

[26] Kobos P.H., Drennen T.E., Outkin A.V., Webb E.K., Paap S.M., Wiryadinata S. (Dec. 2020). “Techno-Economic Analysis: Best Practices and Assessment Tools,” Sandia National Lab. (SNL-CA), Livermore, CA (United States); Sandia National Lab. (SNL-NM), Albuquerque, NM (United States); Hobart and William Smith Colleges, Geneva, NY (United States), SAND-2020-13473.

[27] Scown C.D., Baral N.R., Yang M., Vora N., Huntington T. (Feb. 2021). Technoeconomic analysis for biofuels and bioproducts. Curr. Opin. Biotechnol..

[28] Rechberger H., Brunner P.H. (2016).

[29] Mealing V., Landis A. (Feb. 2023). A life cycle assessment of guar agriculture. Clean Technol. Environ. Policy.

[30] FHWA, “LCA Pave Tool,” Pavements - Federal Highway Administration. Accessed: December. 5, 2023. [Online]. Available: https://www.fhwa.dot.gov/pavement/lcatool/.

[31] DOT, “Infrastructure Voluntary Evaluation Sustainability Tool (INVEST) | US Department of Transportation,” U.S. Department of Transportation. Accessed: December. 05, 2023. [Online]. Available: https://www.transportation.gov/grants/dot-navigator/infrastructure-voluntary-evaluation-sustainability-tool-invest.

[32] ARRB and NACOE, “SUSTAINABILITY - Sustainability Assessment Tool For Pavements (SAT4P),” NACOE. Accesse : December. 5, 2023. [Online]. Available: https://www.nacoe.com.au/projects/pavements-sustainability-assessment-tool/.

[33] Greenroads Foundation, “The Greenroads Rating System,” Sustainable Transport Council Greenroads Foundation. Acces ed: December. 5, 2023. [Online]. Available: https://www.transportcouncil.org/publications.

[34] ISI, “Use Envision - Institute for Sustainable Infrastructure,” Institute for Sustainable Infrastructure (ISI). Acces ed: December. 5, 2023. [Online]. Available: https://sustainableinfrastructure.org/envision/use-envision/.

[35] ISC, “Infrastructure Sustainability (IS) Rating Scheme,” Infrastructure Sustain. Council (ISC). Acces sed: December. 5, 2023. [Online]. Available: https://www.iscouncil.org/is-ratings/.

[36] Meijer J. (Nov. 2021). LCA Pave: A Tool to Assess Environmental Impacts of Pavement Material and Design Decisions - Underlying Methodology and Assumptions.

[37] Mattinzioli T., Sol-Sánchez M., Martínez G., Rubio-Gámez M. (Dec. 2020). A critical review of roadway sustainable rating systems. Sustain. Cities Soc..

[38] Anderson J.L., Muench S.T. (Jan. 2013). Sustainability trends measured by the Greenroads rating system. Transport. Res. Rec..

[39] Scrivener K.L., John V.M., Gartner E.M. (Dec. 2018). Eco-efficient cements: potential economically viable solutions for a low-CO2 cement-based materials industry. Cement Concr. Res..

[40] Ley M.T., Lloyd Z., Kang S., Cook D. (Sep. 2021). Illinois center for transportation. Oklahoma State University, “Concrete Pavement Mixtures With High Supplementary Cementitious Materials Content.

[41] Jahangirnejad S., Van Dam T., Morian D., Smith K., Perera R., Tyson S. (Jan. 2013). Blast furnace slag as sustainable material in concrete pavements. Transport. Res. Rec..

[42] Juenger M.C.G., Snellings R., Bernal S.A. (Aug. 2019). Supplementary cementitious materials: new sources, characterization, and performance insights. Cement Concr. Res..

[43] Hossain Md U., Poon C.S., Dong Y.H., Xuan D. (Feb. 2018). Evaluation of environmental impact distribution methods for supplementary cementitious materials. Renew. Sustain. Energy Rev..

[44] Hanna K., Morcous G., Tadros M.K. (Apr. 2014). Effect of supplementary cementitious materials on the performance of concrete pavement. J. Mater. Civ. Eng..

[45] Snellings R. (Aug. 2016). Assessing, understanding and unlocking supplementary cementitious materials. RILEM Technical Letters.

[46] Nie S. (Feb. 2022). Analysis of theoretical carbon dioxide emissions from cement production: methodology and application. J. Clean. Prod..

[47] Giesekam J., Barrett J.R., Taylor P. (May 2016). Construction sector views on low carbon building materials. Build. Res. Inf..

[48] Kisku N., Joshi H., Ansari M., Panda S.K., Nayak S., Dutta S.C. (Jan. 2017). A critical review and assessment for usage of recycled aggregate as sustainable construction material. Construct. Build. Mater..

[49] Pradyumna T.A., Mittal A., Jain P.K. (Dec. 2013). Characterization of reclaimed asphalt pavement (RAP) for use in bituminous road construction. Proc. - Soc. Behav. Sci..

[50] Chai L., Monismith C.L., Harvey J.T. (Oct. 2009). Re-Cementation of Crushed Material in Pavement Bases.

[51] Chen J.-S., Wei S.-H. (Dec. 2016). Engineering properties and performance of asphalt mixtures incorporating steel slag. Construct. Build. Mater..

[52] Huang Y., Bird R.N., Heidrich O. (Nov. 2007). A review of the use of recycled solid waste materials in asphalt pavements. Resour. Conserv. Recycl..

[53] Basar H.M., Deveci Aksoy N. (Oct. 2012). The effect of waste foundry sand (WFS) as partial replacement of sand on the mechanical, leaching and micro-structural characteristics of ready-mixed concrete. Construct. Build. Mater..

[54] Siddique R., Singh G. (Sep. 2011). Utilization of waste foundry sand (WFS) in concrete manufacturing. Resour. Conserv. Recycl..

[55] Jamshidi A., Kurumisawa K., Nawa T., Igarashi T. (Oct. 2016). Performance of pavements incorporating waste glass: the current state of the art. Renew. Sustain. Energy Rev..

[56] Arulrajah A., Jegatheesan P., T A., Bo M. (Oct. 2011). Geotechnical properties of recycled crushed brick in pavement applications. J. Mater. Civ. Eng..

[57] Milad A., Mohd Taib A., Ahmeda A., Solla M., Md Yusoff N.I. (May 2020). A review of the use of reclaimed asphalt pavement for road paving applications. J. Teknologi.

[58] Tarsi G., Tataranni P., Sangiorgi C. (Jan. 2020). The challenges of using reclaimed asphalt pavement for new asphalt mixtures: a review. Materials.

[59] Novak J., Kohoutková A., Křístek V., Vodička J. (Sep. 2017). Precast concrete pavement – systems and performance review. IOP Conf. Ser. Mater. Sci. Eng..

[60] Ashtiani R.S., de Haro G. (Oct. 2014). “Performance Determination of Precast Concrete Slabs Used for the Repair of Rigid Pavements,”.

[61] Khayat K.H., Valipour M. (Aug. 2014). https://rosap.ntl.bts.gov/view/dot/27955.

[62] Bache H.H. (Dec. 1981). Densified cement ultra-fine particle-based materials. https://www.osti.gov/etdeweb/biblio/10168064.

[63] Ghafari E., Costa H., Júlio E. (Dec. 2015). Critical review on eco-efficient ultra high performance concrete enhanced with nano-materials. Construct. Build. Mater..

[64] Richard P., Cheyrezy M. (Oct. 1995). Composition of reactive powder concretes. Cement Concr. Res..

[65] Van Tuan N., Ye G., van Breugel K., Fraaij A.L.A., Bui D.D. (Apr. 2011). The study of using rice husk ash to produce ultra high performance concrete. Construct. Build. Mater..

[66] Ahmed T., Elchalakani M., Basarir H., Karrech A., Sadrossadat E., Yang B. (Oct. 2021). Development of ECO-UHPC utilizing gold mine tailings as quartz sand alternative. Cleaner Eng. Technol..

[67] Altreuther B., Maennel M. (Sep. 2018). 2018 Joint Conference - Acoustics.

[68] Chao S.-H. (Dec. 2018). https://repository.lsu.edu/transet_pubs/23.

[69] de Larrard F., Sedran T. (Aug. 2011). 9th International Symposium on High Performance Concrete : Design, Verification and Utilization.

[70] Sheheryar M., Rehan R., Nehdi M.L. (Feb. 2021). Estimating CO2 emission savings from ultrahigh performance concrete: a system dynamics approach. Materials.

[71] Busari A.A., Akinmusuru J.O., Dahunsi B.I.O., Ogbiye A.S., Okeniyi J.O. (Jul. 2017). Self-compacting concrete in pavement construction: strength grouping of some selected brands of cements. Energy Proc..

[72] Hesami S., Salehi Hikouei I., Emadi S.A.A. (Oct. 2016). Mechanical behavior of self-compacting concrete pavements incorporating recycled tire rubber crumb and reinforced with polypropylene fiber. J. Clean. Prod..

[73] Rudnicki T. (Jan. 2021). Functional method of designing self-compacting concrete. Materials.

[74] Singh R.B., Debbarma S., Kumar N., Singh S. (Jan. 2021). Hardened state behaviour of self-compacting concrete pavement mixes containing alternative aggregates and secondary binders. Construct. Build. Mater..

[75] Gesoglu M., Güneyisi E., Öz H.Ö., Taha I., Yasemin M.T. (Nov. 2015). Failure characteristics of self-compacting concretes made with recycled aggregates. Construct. Build. Mater..

[76] Grdic Z.J., Toplicic-Curcic G.A., Despotovic I.M., Ristic N.S. (Jul. 2010). Properties of self-compacting concrete prepared with coarse recycled concrete aggregate. Construct. Build. Mater..

[77] Herbudiman B., Saptaji A.M. (Jan. 2013). Self-compacting concrete with recycled traditional roof tile powder. Procedia Eng..

[78] Kou S.C., Poon C.S. (Oct. 2009). Properties of self-compacting concrete prepared with coarse and fine recycled concrete aggregates. Cement Concr. Compos..

[79] Kou S.C., Poon C.S. (Feb. 2009). Properties of self-compacting concrete prepared with recycled glass aggregate. Cement Concr. Compos..

[80] Manzi S., Mazzotti C., Chiara Bignozzi M. (Dec. 2017). Self-compacting concrete with recycled concrete aggregate: study of the long-term properties. Construct. Build. Mater..

[81] Revilla-Cuesta V., Skaf M., Faleschini F., Manso J.M., Ortega-López V. (Jul. 2020). Self-compacting concrete manufactured with recycled concrete aggregate: an overview. J. Clean. Prod..

[82] Santos S., da Silva P.R., de Brito J. (Mar. 2019). Self-compacting concrete with recycled aggregates – a literature review. J. Build. Eng..

[83] Wang J., Zhou J., Kangwa J., Xu Y., Jin R. (2023). Multi-Functional Concrete with Recycled Aggregates.

[84] Jr P., J T. (Jul. 1998). Concrete PAVEMENTS--PAST, present, and future. Public Roads.

[85] Jain S., Singh B. (Jan. 2021). Cold mix asphalt: an overview. J. Clean. Prod..

[86] Cheraghian G. (Sep. 2020). Warm mix asphalt technology: an up to date review. J. Clean. Prod..

[87] Hurley G.C., Prowell B.D. (Jun. 2005).

[88] Rubio M.C., Martínez G., Baena L., Moreno F. (Mar. 2012). Warm mix asphalt: an overview. J. Clean. Prod..

[89] Milad A. (Jan. 2022). A comparative review of hot and warm mix asphalt technologies from environmental and economic perspectives: towards a sustainable asphalt pavement. Int. J. Environ. Res. Publ. Health.

[90] Nithinchary J., Dhandapani B.P., Mullapudi R.S. (Feb. 2024). Application of warm mix technology - design and performance characteristics: review and way forward. Construct. Build. Mater..

[91] Dash S.S., Chandrappa A.K., Sahoo U.C. (Jan. 2022). Design and performance of cold mix asphalt – a review. Construct. Build. Mater..

[92] Mills-Beale J., You Z., Fini E., Zada B., Lee C.H., Yap Y.K. (Feb. 2014). Aging influence on rheology properties of petroleum-based asphalt modified with biobinder. J. Mater. Civ. Eng..

[93] Fini E.H., Yang S.-H., Xiu S. (2010). Presented at the Transportation Research Board 89th Annual MeetingTransportation Research Board.

[94] Jose S., Bhaskar T. (2015).

[95] Lei Z., Bahia H., Yi-qiu T. (Jul. 2015). Effect of bio-based and refined waste oil modifiers on low temperature performance of asphalt binders. Construct. Build. Mater..

[96] Warith K.A., Khedr S. (Dec. 2013). Investigating a natural plant-based bio binder and cement dust mix as a bitumen substitute in flexible pavements. Adv. Civ. Eng. Matls..

[97] Yang X., You Z., Dai Q. (Jul. 2013). Performance evaluation of asphalt binder modified by bio-oil generated from waste wood resources. Int. J. Pavement Res. Technol..

[98] Su N., Xiao F., Wang J., Cong L., Amirkhanian S. (Sep. 2018). Productions and applications of bio-asphalts – a review. Construct. Build. Mater..

[99] Zhang Z., Fang Y., Yang J., Li X. (Apr. 2022). A comprehensive review of bio-oil, bio-binder and bio-asphalt materials: their source, composition, preparation and performance. J. Traffic Transport. Eng..

[100] Weidong C., Xiaobo Z., Qi X. (2014). Advances in bio-asphalt research. Petroleum Asphalt.

[101] Praticò F.G., Perri G., De Rose M., Vaiana R. (Oct. 2023). Comparing bio-binders, rubberised asphalts, and traditional pavement technologies. Construct. Build. Mater..

[102] Dhar P. (Jul. 2023). Asphalt that's safer for humans and the environment. C&EN Global Enterp.

[103] T. Grant, “New asphalt binder alternative is less toxic, more sustainable than conventional blend,” ASU Newsl. Accessed: October. 22, 2023. [Online]. Available: https://news.asu.edu/20230918-solutions-new-asphalt-binder-alternative-less-toxic-more-sustainable-conventional-blend.

[104] Rahman M.T., Mohajerani A., Giustozzi F. (Jan. 2020). Recycling of waste materials for asphalt concrete and bitumen: a review. Materials.

[105] Kalantar Z.N., Karim M.R., Mahrez A. (Aug. 2012). A review of using waste and virgin polymer in pavement. Construct. Build. Mater..

[106] Santamarina J.C. (Jan. 2014). Georgia Institute of Technology, Georgia Department of Transportation Office of Research, U.S. Department of Transportation Federal Highway Administration.

[107] Jiang X. (Sep. 2022). Evaluating the performance of inverted pavement structure using the accelerated pavement test (APT). Construct. Build. Mater..

[108] Ahmed I. (Dec. 2021). A mechanistic approach to evaluate the fatigue life of inverted pavements. Construct. Build. Mater..

[109] Avellaneda D.D.C. (2010). “Inverted Base Pavement Structures,”.

[110] Ishai I., Comparative economic-engineering evaluation of concrete block pavements, Road Mater. Pavement Des. 4 (3) (Jan. 2003) 251-268, doi: 10.1080/14680629.2003.9689948.

[111] Pompigna A., Mauro R. (Jan. 2022). Smart roads: a state of the art of highways innovations in the Smart Age. Eng. Sci. Technol., an Int. J..

[112] Singh R. (Nov. 2021). Highway 4.0: digitalization of highways for vulnerable road safety development with intelligent IoT sensors and machine learning. Saf. Sci..

[113] Shtayat A., Moridpour S., Best B., Shroff A., Raol D. (Oct. 2020). A review of monitoring systems of pavement condition in paved and unpaved roads. J. Traffic Transport. Eng..

[114] Wang J., Han Y., Cao Z., Xu X., Zhang J., Xiao F. (Oct. 2023). Applications of optical fiber sensor in pavement Engineering: a review. Construct. Build. Mater..

[115] Birgin H.B., D'Alessandro A., Laflamme S., Ubertini F. (Jul. 2021). Innovative carbon-doped composite pavements with sensing capability and low environmental impact for multifunctional infrastructures. J. Composites Sci..

[116] Birgin H.B., D'Alessandro A., Favaro M., Sangiorgi C., Laflamme S., Ubertini F. (Jun. 2022). Field investigation of novel self-sensing asphalt pavement for weigh-in-motion sensing. Smart Mater. Struct..

[117] Tabakovic A., Schlangen E. (2015).

[118] Anupam B.R., Sahoo U.C., Chandrappa A.K. (Feb. 2022). A methodological review on self-healing asphalt pavements. Construct. Build. Mater..

[119] Bazin P., Saunier J. (Jan. 1967). Presented at the International Conference on the Structural Design of Asphalt Pavements.

[120] Hajj R., Garg N., Doehring J., Vyas A., Asadi B., Lu Y. (Jan. 2023). “Using Microcapsules and Bacteria for Self-Healing in Rigid and Flexible Pavements,”.

[121] Gonzalez-Torre I., Norambuena-Contreras J. (Oct. 2020). Recent advances on self-healing of bituminous materials by the action of encapsulated rejuvenators. Construct. Build. Mater..

[122] Qiu J., Ven M., Wu S., Yu J., Molenaar A. (Jun. 2009). Investigating the self healing capability of bituminous binders. Road Mater. Pavement Des..

[123] Ganjei M.A., Aflaki E. (Jan. 2019). Application of nano-silica and styrene-butadiene-styrene to improve asphalt mixture self healing. Int. J. Pavement Eng..

[124] Tabatabaee N., Shafiee M.H. (2012). 7th RILEM International Conference on Cracking in Pavements, A. Scarpas, N. Kringos, I. Al-Qadi, and L. A., Eds., in RILEM Bookseries.

[125] Norambuena-Contreras J., Garcia A. (Sep. 2016). Self-healing of asphalt mixture by microwave and induction heating. Mater. Des..

[126] Leiva-Padilla P., Moreno-Navarro F., Iglesias-Salto G., Rubio-Gamez M.C. (Dec. 2020). Recovery capacity of electroconductive asphalt mortars under the influence of magnetic fields. Mater. Today Commun..

[127] Leiva-Padilla P., Moreno-Navarro F., Iglesias G., Rubio-Gamez M.C. (Mar. 2020). A review of the contribution of mechanomutable asphalt materials towards addressing the upcoming challenges of asphalt pavements. Infrastructure.

[128] Moreno-Navarro F., Iglesias G.R., Rubio-Gámez M.C. (Nov. 2015). Development of mechanomutable asphalt binders for the construction of smart pavements. Mater. Des..

[129] Choi Y. (Dec. 2022). Development of the Austroads Rejuvenator Evaluation Protocol.

[130] He Y., Xiong K., Zhang J., Guo F., Li Y., Hu Q. (Mar. 2024). A state-of-the-art review and prospectives on the self-healing repair technology for asphalt materials. Construct. Build. Mater..

[131] Jonkers H.M., Thijssen A., Muyzer G., Copuroglu O., Schlangen E. (Feb. 2010). Application of bacteria as self-healing agent for the development of sustainable concrete. Ecol. Eng..

[132] Luhar S., Luhar I., Shaikh F.U.A. (Jan. 2022). A review on the performance evaluation of autonomous self-healing bacterial concrete: mechanisms, strength, durability, and microstructural properties. J. Composites Sci..

[133] Xue C., Li W., Li J., Tam V.W.Y., Ye G. (2019). A review study on encapsulation-based self-healing for cementitious materials. Struct. Concr..

[134] Rosewitz J.A., Wang S., Scarlata S.F., Rahbar N. (Jun. 2021). An enzymatic self-healing cementitious material. Appl. Mater. Today.

[135] Huang H., Ye G., Qian C., Schlangen E. (Feb. 2016). Self-healing in cementitious materials: materials, methods and service conditions. Mater. Des..

[136] Chen J. (Dec. 2021). New innovations in pavement materials and engineering: a review on pavement engineering research 2021. J. Traffic Transport. Eng..

[137] Dong J., Meng W., Liu Y., Ti J. (Jan. 2021). A framework of pavement management system based on IoT and big data. Adv. Eng. Inf..

[138] Malik R. (Jul. 2019). Mapping and deep analysis of vehicle-to-infrastructure communication systems: coherent taxonomy, datasets, evaluation and performance measurements, motivations, open challenges, recommendations, and methodological aspects. IEEE Access.

[139] Malik R.Q., Ramli Khairun N., Kareem Z.H., Habelalmatee M.I., Abbas H. (Sep. 2020). 2020 3rd International Conference on Engineering Technology and its Applications (IICETA).

[140] Ahmad S., Abdul Mujeebu M., Farooqi Mohd A. (2019). Energy harvesting from pavements and roadways: a comprehensive review of technologies, materials, and challenges. Int. J. Energy Res..

[141] Al-Qadami E.H.H., Mustaffa Z., Al-Atroush M.E. (Jan. 2022). Evaluation of the pavement geothermal energy harvesting technologies towards sustainability and renewable energy. Energies.

[142] Dezfooli A.S., Nejad F.M., Zakeri H., Kazemifard S. (Jun. 2017). Solar pavement: a new emerging technology. Sol. Energy.

[143] Wang H., Jasim A., Chen X. (Feb. 2018). Energy harvesting technologies in roadway and bridge for different applications – a comprehensive review. Appl. Energy.

[144] Zhao H.D., Ling J.M., Fu P.C. (2013). A review of harvesting green energy from road. Adv. Mater. Res..

[145] Renoald M.A.J., Hemalatha V., Punitha R., Sasikala M., Sasikala M.E. (2016). Solar roadways-the future rebuilding infrastructure and economy. Int. J. Electron. Eng. Res..

[146] Hu H., Vizzari D., Zha X., Roberts R. (Dec. 2021). Solar pavements: a critical review. Renew. Sustain. Energy Rev..

[147] Nordmann T. (2001). Sixteenth European Photovoltaic Solar Energy Conference.

[148] Paudel A., Hirsch A., “Potential impacts of solar arrays on highway environment, Safety Oper.,” Colorado Dep. Transport. Appl. Res. Innov. Branch in Cooperation with the Fed. Highway Admin. (Oct. 2015) Colorado State University- Pueblo, CDOT-2015-08 https://www.codot.gov/programs/research/pdfs/2015-research-reports/solar-arrays. (Accessed 23 October 2023).

[149] García A., Partl M.N. (Apr. 2014). How to transform an asphalt concrete pavement into a solar turbine. Appl. Energy.

[150] O. Us Epa, “Using Cool Pavements to Reduce Heat Islands,” United States Environmental Protection Agency. Accessed: June. 8, 2023. [Online]. Available: https://www.epa.gov/heatislands/using-cool-pavements-reduce-heat-islands.

[151] Akbari H., Pomerantz M., Taha H. (Jan. 2001). Cool surfaces and shade trees to reduce energy use and improve air quality in urban areas. Sol. Energy.

[152] Anupam B.R., Sahoo U.C., Chandrappa A.K., Rath P. (Sep. 2021). Emerging technologies in cool pavements: a review. Construct. Build. Mater..

[153] Lin T.-P., Ho Y.-F., Huang Y.-S. (Dec. 2007). Seasonal effect of pavement on outdoor thermal environments in subtropical Taiwan. Build. Environ..

[154] Yang L., Qian F., Song D.-X., Zheng K.-J. (Jan. 2016). Research on urban heat-island effect. Procedia Eng..

[155] O. Us Epa, “Heat Island Impacts,” United States Environmental Protection Agency. Asccessed: June. 28, 2023. [Online]. Available: https://www.epa.gov/heatislands/heat-island-impacts.

[156] O. US EPA, “Heat Islands and Equity,” United States Environmental Protection Agency. Accessesd: June. 28, 2023. [Online]. Available: https://www.epa.gov/heatislands/heat-islands-and-equity.

[157] Garton D., Hulme S., Bodin D. (Aug. 2021). Australian Road Research Board (ARRB), Queensland Department of Transport and Main Roads' and the National Asset Centre of Excellence (NACOE), State of Queensland, Australia, ARRB Project No.: 015737.

[158] Jiménez J.R., Ayuso J., Agrela F., López M., Galvín A.P. (Jan. 2012). Utilisation of unbound recycled aggregates from selected CDW in unpaved rural roads. Resour. Conserv. Recycl..

[159] Trochez J., Grenfell J., Harrison J. (Feb. 2021). Australian Road Research Board (ARRB), Queensland Department of Transport and Main Roads' and the National Asset Centre of Excellence (NACOE), State of Queensland, Australia, ARRB Project No.

[160] Huber S., Henzinger C., Heyer D. (Mar. 2020). Influence of water and frost on the performance of natural and recycled materials used in unpaved roads and road shoulders. Transport. Geotech..

[161] Jiménez J.R., Ayuso J., Galvín A.P., López M., Agrela F. (Sep. 2012). Use of mixed recycled aggregates with a low embodied energy from non-selected CDW in unpaved rural roads. Construct. Build. Mater..

[162] Johannessen D., Xu A., Garton D., Rae S., Roberts W. (Oct. 2021). Australian Road Research Board (ARRB), Queensland Department of Transport and Main Roads' and the National Asset Centre of Excellence (NACOE).

[163] TMR (Sep. 2020). Technical Note TN193: use of recycled materials in road construction. The State of Queensland (Dep. Transport and Main Roads), Queensland, Australia.

[164] Giroud J.P., Han J. (Aug. 2004). Design method for geogrid-reinforced unpaved roads. I. Development of design method. J. Geotech. Geoenviron. Eng..

[165] Palmeira E.M., Antunes L.G.S. (Dec. 2010). Large scale tests on geosynthetic reinforced unpaved roads subjected to surface maintenance. Geotext. Geomembranes.

[166] Singh M., Trivedi A., Shukla S.K. (Aug. 2022). Evaluation of geosynthetic reinforcement in unpaved road using moving wheel load test. Geotext. Geomembranes.

[167] Biswas A., Krishna A.M. (May 2017). Geocell-reinforced foundation systems: a critical review. Int. J. of Geosynth. and Ground Eng..

[168] Birajdar S.J., Iraganti J.G., Mungapatil P.A., Jamadar S.V., Gajul P.U., Kshirsagar R.S. (Jul. 2021). Soil stabilization by using geocell. Int. J. Adv. Eng. Manag. (IJAEM).

[169] Inti S., Tandon V. (Mar. 2022).

[170] Thom N., Dawson A. (Jan. 2019). Sustainable road design: promoting recycling and non-conventional materials. Sustainability.

[171] Troeger J., Widyatmoko D. (2012).

[172] Miljković M., Radenberg M. (Jan. 2016). Effect of compaction energy on physical and mechanical performance of bitumen emulsion mortar. Mater. Struct..

[173] Perić D., Bartley P.A., Davis L., Uzer A.U., Gürer C. (Mar. 2016). Assessment of sand stabilization potential of a plant-derived biomass. Sci. Eng. Compos. Mater..

[174] Uzer A.U. (Jan. 2015). Use of biofuel Co-product for pavement geo-materials stabilization. Procedia Eng..

[175] Uzer A.U. (Nov. 2023). Evaluation of shear stress in soils stabilized with biofuel Co-products via regression analysis methods. Buildings.

[176] Yang B., Zhang Y., Ceylan H., Kim S., Gopalakrishnan K. (Dec. 2018). Assessment of soils stabilized with lignin-based byproducts. Transport. Geotech..

[177] Zhang T., Cai G., Liu S., Puppala A.J. (2014). Stabilization of silt using a lignin-based bioenergy coproduct. Presented at the Transport. Res. Board 93rd Annual Meet.Transport. Res. Board.

[178] Yao H. (2022). Review on applications of lignin in pavement engineering: a recent survey. Front. Mater..

[179] Vaiana R., Oliviero Rossi C., Perri G. (Jan. 2021). An eco-sustainable stabilization of clayey road subgrades by lignin treatment: an overview and a comparative experimental investigation. Appl. Sci..

[180] Kestler M.A. (Mar. 2009).

[181] Ceylan H., Gopalakrishnan K., Kim S. (Jan. 2010). Soil stabilization with bioenergy coproduct. Transport. Res. Rec..

[182] Kim S., Gopalakrishnan K., Ceylan H. (Nov. 2012). Moisture susceptibility of subgrade soils stabilized by lignin-based renewable energy coproduct. J. Transport. Eng..

[183] Santoni R.L., Tingle J.S., Webster S.L. (Jan. 2002). Stabilization of silty sand with nontraditional additives. Transport. Res. Rec..

[184] Tingle J.S., Santoni R.L. (Jan. 2003). Stabilization of clay soils with nontraditional additives. Transport. Res. Rec..

[185] Uzer A.U. (Jul. 2016). Evaluation of freezing-thawing cycles for foundation soil stabilization. Soil Mech. Found. Eng..

[186] Zhang T., Cai G., Liu S. (Jan. 2017). Application of lignin-based by-product stabilized silty soil in highway subgrade: a field investigation. J. Clean. Prod..

[187] Zhang T., Yang Y.-L., Liu S.-Y. (Aug. 2020). Application of biomass by-product lignin stabilized soils as sustainable Geomaterials: a review. Sci. Total Environ..

[188] Taher S.A., Alyousify S., Hassan H.J.A. (2020). Comparative study of using flexible and rigid pavements for roads: a review study. J. Donghua Univ..

[189] Wimsatt A.J., Krugler P.E., Freeman T.J., Chang-Albitres C.M., Scullion T., Valdovinos M.B. (2009). “Considerations for Rigid vs. Flexible Pavement Designs when Allowed as Alternate Bids: Technical Report,”.

[190] Mohod M.V., Kadam K.N. (2016). A comparative study on rigid and flexible pavement: a review. IOSR J. Mech. Civ. Eng..

[191] Ketema Y., Quezon E.T., Kebede G. (Oct. 2016). Cost and benefit analysis of rigid and flexible pavement: a case study at chancho –Derba-Becho road project. Int. J. Sci. Eng. Res..

[192] Mehta P.K., Monteiro P.J.M. (2001).

[193] Wbcsd I. (2009). International Energy Agency) IEA Technology Roadmaps.

[194] Fu H., Wang C., Niu L., Yang G., Liu L. (Sep. 2022). Composition optimisation and performance evaluation of waterborne epoxy resin emulsified asphalt tack coat binder for pavement. Int. J. Pavement Eng..

[195] Wang C., Li Q., Wang K.C.P., Sun X., Wang X. (Mar. 2017). Emission reduction performance of modified hot mix asphalt mixtures. Adv. Mater. Sci. Eng..

[196] Wang C., Wang M., Chen Q., Zhang L. (Jan. 2022). Basic performance and asphalt smoke absorption effect of environment-friendly asphalt to improve pavement construction environment. J. Clean. Prod..

[197] Tutu K.A., Tuffour Y.A. (2016). Warm-mix asphalt and pavement sustainability: a review. Open J. Civ. Eng..

[198] Huang B., Li G., Vukosavljevic D., Shu X., Egan B.K. (Jan. 2005). Laboratory investigation of mixing hot-mix asphalt with reclaimed asphalt pavement. Transport. Res. Rec..

[199] Kamil Shanbara H., Ruddock F., Atherton W. (2018). Science and Technology behind Nanoemulsions.

[200] Ling C., Hanz A., Bahia H. (Feb. 2016). Measuring moisture susceptibility of Cold Mix Asphalt with a modified boiling test based on digital imaging. Construct. Build. Mater..

[201] Rusbintardjo G., Hainin Mohd R., Yusoff N. I. Md (Dec. 2013). Fundamental and rheological properties of oil palm fruit ash modified bitumen. Construct. Build. Mater..

[202] Mukhopadhyay S.C., Suryadevara N.K., Mukhopadhyay S.C. (2014). Internet of Things: Challenges and Opportunities.

[203] Zhao H., Wu D. (Oct. 2015).

[204] (2019). Sustainable Mobility for All, Global Roadmap of Action toward Sustainable Mobility.

[205] African Development Bank (2014).

[206] Parvej S., Naik D.L., Sajid H.U., Kiran R., Huang Y., Thanki N. (Jan. 2021). Fugitive dust suppression in unpaved roads: state of the art research review. Sustainability.

[207] Bureau of Transportation Statistics (2015). “National Transportation Statistics,” Research and Innovative Technology Administration, Washington DC, USA.

[208] Paige-Green P., Verhaeghe B., Head M. (Sep. 2016).

[209] Alzubaidi H., Magnusson R. (Jan. 2002). Deterioration and rating of gravel roads. Road Mater. Pavement Des..

[210] Eaton R.A. (1992).

[211] Schnebele E., Tanyu B.F., Cervone G., Waters N. (Jun. 2015). Review of remote sensing methodologies for pavement management and assessment. Eur. Transp. Res. Rev..

[212] Miller J.S., Bellinger W.Y. (May 2014). “Distress Identification Manual for the Long-Term Pavement Performance Program (Fifth Revised Edition),” FHWA-HRT-13-092.

[213] Riverson J.D.N., Sinha K.C., Scholer C.F., Anderson V.L. (1987). Evaluation of subjective rating of unpaved county roads in Indiana. Transport. Res. Rec..

[214] Zhang C., Elaksher A. (2012). An unmanned aerial vehicle-based imaging system for 3D measurement of unpaved road surface Distresses1. Comput. Aided Civ. Infrastruct. Eng..

[215] Jameson G. (2019). Austroads.

[216] Tan E.H., Zahran E.M.M., Tan S.J. (Oct. 2020). A review of chemical stabilisation in road construction. IOP Conf. Ser. Mater. Sci. Eng..

[217] Hopkins C., Cameron D., Rahman M.M. (Jul. 2023). Field trials of unbound granular pavements treated with an insoluble dry powdered polymer. Transport. Geotech..

[218] Huang J., Kogbara R.B., Hariharan N., Masad E.A., Little D.N. (Oct. 2021). A state-of-the-art review of polymers used in soil stabilization. Construct. Build. Mater..

[219] Pooni J., Robert D., Giustozzi F., Setunge S., Venkatesan S. (2022). Stabilisation of expansive soils subjected to moisture fluctuations in unsealed road pavements. Int. J. Pavement Eng..

[220] Zhalehjoo N., Grenfell J. (Oct. 2023). Presented at the National Transport Research Organization International Technical Conference.

[221] Xiao F., Yao S., Wang J., Li X., Amirkhanian S. (Aug. 2018). A literature review on cold recycling technology of asphalt pavement. Construct. Build. Mater..

